# Octahedral small virus-like particles of dengue virus type 2

**DOI:** 10.1128/jvi.01809-24

**Published:** 2024-12-31

**Authors:** Adam Johnson, Martín Dodes Traian, Richard M. Walsh, Simon Jenni, Stephen C. Harrison

**Affiliations:** 1 Department of Biological Chemistry and Molecular Pharmacology, Harvard Medical School 189702 , Boston, Massachusetts, USA; 2 Laboratory of Molecular Medicine, Boston Children’s Hospital, Boston, Massachusetts, USA; 3 Howard Hughes Medical Institute, Harvard Medical School 1811 , Boston, Massachusetts, USA; Wake Forest University School of Medicine, Winston-Salem, North Carolina, USA

**Keywords:** flavivirus, structure, cryo-EM, vaccine

## Abstract

Flavivirus envelope (E) and precursor M (prM) proteins, when ectopically expressed, assemble into empty, virus-like particles (VLPs). Cleavage of prM to M and loss of the pr fragment converts the VLPs from immature to mature particles, mimicking a similar maturation of authentic virions. Most of the VLPs obtained by prM-E expression are smaller than virions; early, low-resolution cryo-EM studies suggested a simple, 60-subunit, icosahedral organization. We describe here the cryo-EM structure of immature, small VLPs (smVLPs) from dengue virus type 2 and show that they have octahedral rather than icosahedral symmetry. The asymmetric unit of the octahedral particle is an asymmetric trimer of prM-E heterodimers, just as it is on icosahedral immature virions; the full, octahedrally symmetric particle thus has 24 such asymmetric trimers or 72 prM-E heterodimers in all. Cleavage of prM and release of pr generates ovoid, somewhat irregular, mature particles. Previous work has shown that mature smVLPs have fusion properties identical to those of virions, consistent with local, virion-like clustering of 36 E dimers on their surface. The cryo-EM structure and the properties of the smVLPs described here relate directly to ongoing efforts to use them as vaccine immunogens.

**IMPORTANCE:**

Ectopic expression of flavivirus envelope (E) and precursor M (prM) proteins leads to the formation and secretion of empty, virus-like particles (VLPs). We show that a major class of VLPs, of smaller diameter than those of virion size (“small VLPs”: smVLPs), are octahedrally symmetric particles. The known characteristics of immature virions (asymmetric trimers of prM-E heterodimers) allow us to understand the assembly of an octahedral (rather than icosahedral) surface lattice. Cleavage of prM and formation of mature, fusogenic smVLPs yield somewhat irregular, ovoid particles. These observations are directly relevant to proposals for using immunogenic but non-infectious VLPs as components of specific flavivirus vaccines.

## INTRODUCTION

**Fig 1:**
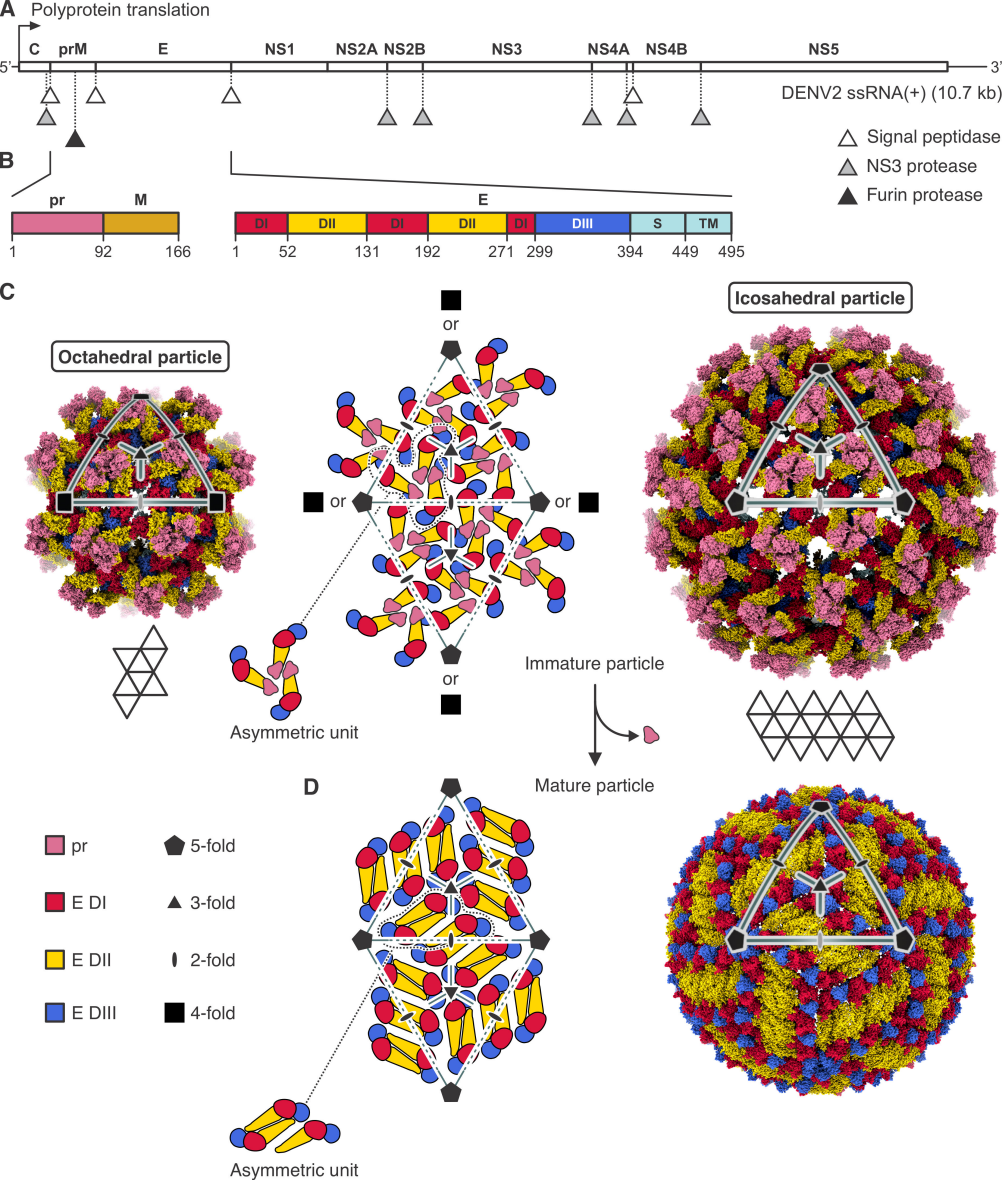
Flavivirus structural organization. (**A**) Flavivirus proteins are produced in the host cell from an approximately 10.7 kb long single-stranded positive sense genome/messenger RNA, ssRNA(+). A polyprotein is translated, which is co-translationally and post-translationally processed by different host and viral proteases (signal peptidase, NS3 protease, furin protease) at positions indicated by triangles. C, (anchored) capsid protein; prM, membrane glycoprotein precursor; E, envelope protein; NS1, nonstructural protein 1; NS2A, nonstructural protein 2A; NS2B, nonstructural protein 2B; NS3, nonstructural protein 3 (protease); NS4A, nonstructural protein 4A; NS4B, nonstructural protein 4B; NS5, nonstructural protein 5 (methyltransferase and RNA-dependent RNA polymerase). (**B**) Primary sequence and domain organization of the prM and E proteins. Residues at domain boundaries are numbered. pr sequence is colored pink, M is brown. E protein domains are labeled I (red), II (yellow), III (blue), S (stem, cyan), and TM (transmembrane region, cyan). prM is cleaved by furin protease after residue 91 (dengue virus type 2 [DENV2] sequence numbering). (**C**) Structural organization of immature flavivirus particles. The middle panel shows schematically the arrangement of prM-E protomers on a pseudohexagonal lattice. Folding of 20 triangular faces around fivefold axes results in icosahedral particles, such as the one shown in the right panel, which is the structure of an immature virion (immature Spondweni virus [SPOV] particle, PDB-ID 6ZQW). Folding of eight triangular faces around fourfold axes results in octahedral particles, such as the one shown in the left panel, which is the structure of the DENV2 small VLP (smVLP) reported here. In both the octahedral and the icosahedral particles, the triangulation number is *T* = 1 with three prM-E protomers per asymmetric unit. Protein domains are colored as in (**B**). (**D**) Structural organization of mature flavivirus particles. Proteolytic cleavage by furin in the TGN, exposure to mildly acidic pH, and release of virions from the host cell with dissociation of the pr protein are accompanied by substantial structural rearrangements of the M and E proteins to generate the mature particle, in which M and E form dimers. The structure of the mature particle has the same icosahedral symmetry as the immature particle (mature SPOV particle, PDB-ID 6ZQV).

Flaviviruses assemble by budding into the endoplasmic reticulum (ER) as immature particles, pass through the secretory pathway, and emerge from the cell as mature, infectious virions. The icosahedrally symmetric, immature particles contain 180 envelope (E) subunits associated with the same number of membrane-protein precursor (prM) subunits and internal, core (C) protein subunits (Fig. 1A). Both E and prM are glycoproteins with C-terminal, transmembrane anchors (Fig. 1B). The conserved E-protein ectodomains (DI, DII, and DIII) connect to the helical-hairpin anchors through a segment traditionally called the “stem” (a term that antedates any structure). The C subunits, on the cytosolic side of the ER membrane, interact with the 10.7 kb, plus-sense, RNA genome and incorporate it into the particle as it assembles and buds into the ER lumen.

Sixty markedly asymmetric clusters of three prM-E protomers cover the surface of an immature particle (Fig. 1C; Fig. S1 and S2) (1–6). Ninety twofold symmetric M-E dimers cover the surface of a mature particle (Fig. 1D; Fig. S1 and S2) (7, 8). The large-scale reorganization of the particle surface, from immature to mature, probably occurs in the trans-Golgi network (TGN), in response to the mildly acidic pH of the TGN lumen (3). The reorganization exposes a furin site on prM; cleavage at that site will allow the release of the N-terminal, “pr” fragment when the particle again reaches neutral pH as it emerges from the infected cell. During infection of a new target cell, exposure to low pH in an endosome triggers a further fusogenic rearrangement of E into post-fusion trimers (9).

Cryo-EM structures have been determined for several mature flavivirus particles, but only three for immature virions at high resolution (5, 6, 10) and one at lower resolution (11). The structures of immature Binjari virus (BinJV) (5) and tick-borne encephalitis virus (TBEV) (10) have defined the previously uncertain connectivity between the pr fragment, which covers the fusion loop of E, and the membrane-associated C-terminal fragment (which becomes “M” in the mature particle). While it is likely that the connectivity in BinJV and TBEV is generally true, as inferred from sequence conservation (Data set S1 to S3 in the supplemental material), density in the structure of Spondweni virus (SPOV) is too poorly defined in the connecting segments to confirm it (6).

Ectopic expression of flavivirus prM-E alone leads to the production and secretion of virus-like particles (VLPs), which undergo the same immature-to-mature transition as virions and which have virion-like fusion properties (12–14). Particles of two or more sizes result. Particles in one class closely resemble virions in size (about 500 Å in diameter) and isometric shape; those in a second class (smVLPs) are smaller with about one-third the number of E protein subunits on their surface (15, 16). The latter predominates in our preparations (14, 17) and in several of those reported in the literature (16). Early work suggested that the smVLPs of TBEV were 60-subunit, icosahedral particles (18). But because icosahedral symmetry for a 60-subunit surface requires symmetric threefold clusters, it is incompatible with the asymmetric threefold clusters now known (from work that came several years later) to be present on immature virions and presumably on the smVLPs. We show here that immature smVLPs of dengue virus type 2 (DENV2), and by extension those of other flaviviruses, are octahedrally symmetric particles, with prM-E asymmetrically clustered as on immature virions. The octahedral asymmetric unit is one asymmetric cluster of three prM-E heterodimers, and thus, there are 72 prM-E heterodimers in total. The transition to mature particles, generated by cleavage *in vitro*, produces somewhat ovoid particles, presumably covered by 36 E dimers.

## RESULTS AND DISCUSSION

**Fig 2:**
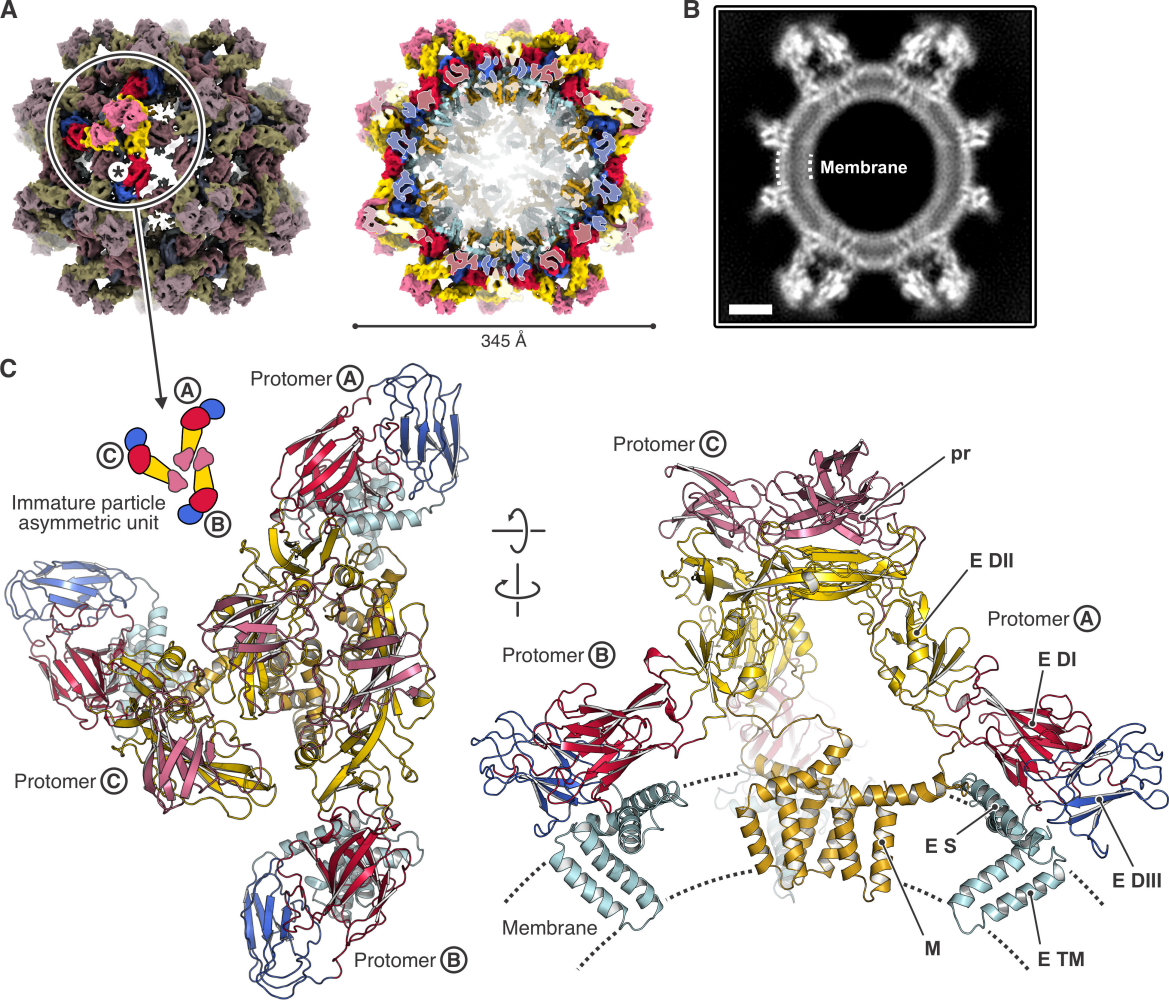
Structure of the DENV2 octahedral small virus-like particle (smVLP). (**A**) Cryo-EM map of the octahedral smVLP at 6.5 Å resolution, viewed along a twofold symmetry axis. prM-E protein domains are colored according to the following scheme: pr, pink; M, brown; E DI, red; E DII, yellow; E DIII, blue; E stem and C-terminal domains, cyan. Left, one asymmetric unit, consisting of an asymmetric trimer of three prM-E protomers (labeled Ⓐ, Ⓑ, and Ⓒ), is highlighted. Asterisk shows position of local twofold axis. Right, the particle is cut to allow inside view of the M and E transmembrane α helices. (**B**) Projection of a 12 Å-thick central slice of the octahedral smVLP cryo-EM map showing density arising from the lipid bilayer. The scale bar corresponds to 50 Å. (**C**) Ribbon representation of the asymmetric trimer structure, viewed from the outside of the particle (left), and viewed from the side (right). The membrane is indicated schematically. Domains are colored as in (**A**).

**Fig 3:**
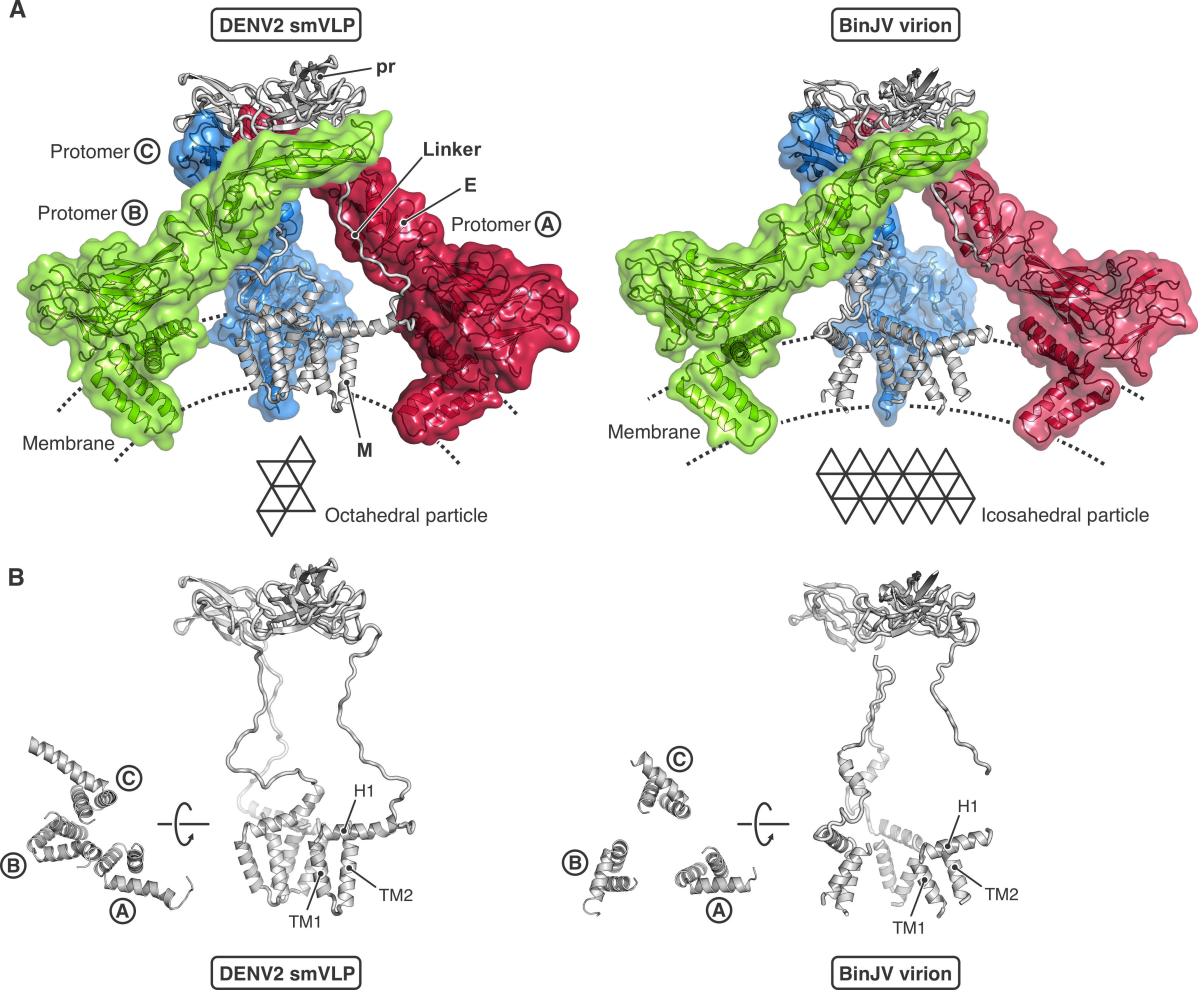
Comparison of the prM-E asymmetric trimer from the octahedral DENV2 smVLP and the icosahedral BinJV immature flavivirus virion. (**A**) DENV2 smVLP (left) and BinJV virion (right, PDB-ID 7L30). The E subunits are shown in transparent surface representation and colored differently: protomer Ⓐ, red; protomer Ⓑ, green; protomer Ⓒ, blue. prM is shown in gray ribbon representation for all three protomers. The octahedral DENV2 smVLP (left) contains 72 prM-E protomers, with three asymmetric trimers within each of the eight triangular faces (3 × 3 × 8 = 72). The icosahedral immature BinJV virion (right) contains 180 prM-E protomers, with three asymmetric trimers within each of the 20 triangular faces (3 × 3 × 20 = 180). (**B**) View of the prM within one asymmetric trimer for the octahedral DENV2 smVLP (left) and the icosahedral immature BinJV virion (right).

We obtained a 3D reconstruction of an immature DENV2 smVLP at 6.5 Å resolution (Fig. 2A and B; Fig. S5A). Focused classification and refinement of a symmetry-expanded particle image stack yielded an improved map of the asymmetric unit (three prM-E heterodimers) at 4.3 Å resolution (Fig. 2C; Fig. S5B). We obtained an AlphaFold 2 structure from the DENV2 prM-E sequence, docked three models of the prM-E heterodimer independently into the three subunits of the asymmetric unit, and manually modeled the linker sequence between pr and M. The model was fit to the locally focused map by domain-wise rigid-body fitting, structure morphing, and real space refinement (Fig. 2C; Fig. S5B and C). The stem and transmembrane segments of E and prM were reasonably well defined in the map, in particular for two of the three protomers (Fig. 3). The sharper curvature of the particle displaced the transmembrane segments from their positions, relative to their ectodomains, in the 180-subunit, immature flavivirions for which subnanometer structures are known (Fig. 3; Data sets S4 and S5). Our map is consistent with the revised prM connectivity seen clearly in the structures of immature BinJV (5) and TBEV (10) in which the three pr domains at the tips of an asymmetric threefold cluster connect directly to the three stem and transmembrane anchors (stem-TMs) clustered around the same pseudo-threefold (Fig. 3A). In our locally refined map, essentially continuous density (weak from residues 215–224, but unambiguously traced for two of the chains and suggested by very weak density for the third) connects each “pr” domain associated with a fusion-loop tip of E with a corresponding stem-TM. One local difference is in the orientation of the prM stem-TM associated with the E subunit marked B in Fig. 3B. In our octahedral smVLP structure, the segment that connects the pr domain and stem-TM of the prM in question allows the B-subunit stem-TM to form an approximately twofold symmetric contact with the stem-TM of prM associated with subunit C—that is, to orient the stem-TM about 180° from its orientation in the full particle (Fig. 3B and C). Moreover, this contact is essentially the same as the twofold contact between M proteins in the mature icosahedral particle. As suggested in early work on dengue virus particles and confirmed by the immature BinJV and TBEV structures, the furin site is inaccessible in the immature particle but becomes exposed on the virion surface during the immature-to-mature lattice transition (Fig. 3B).

**Fig 4:**
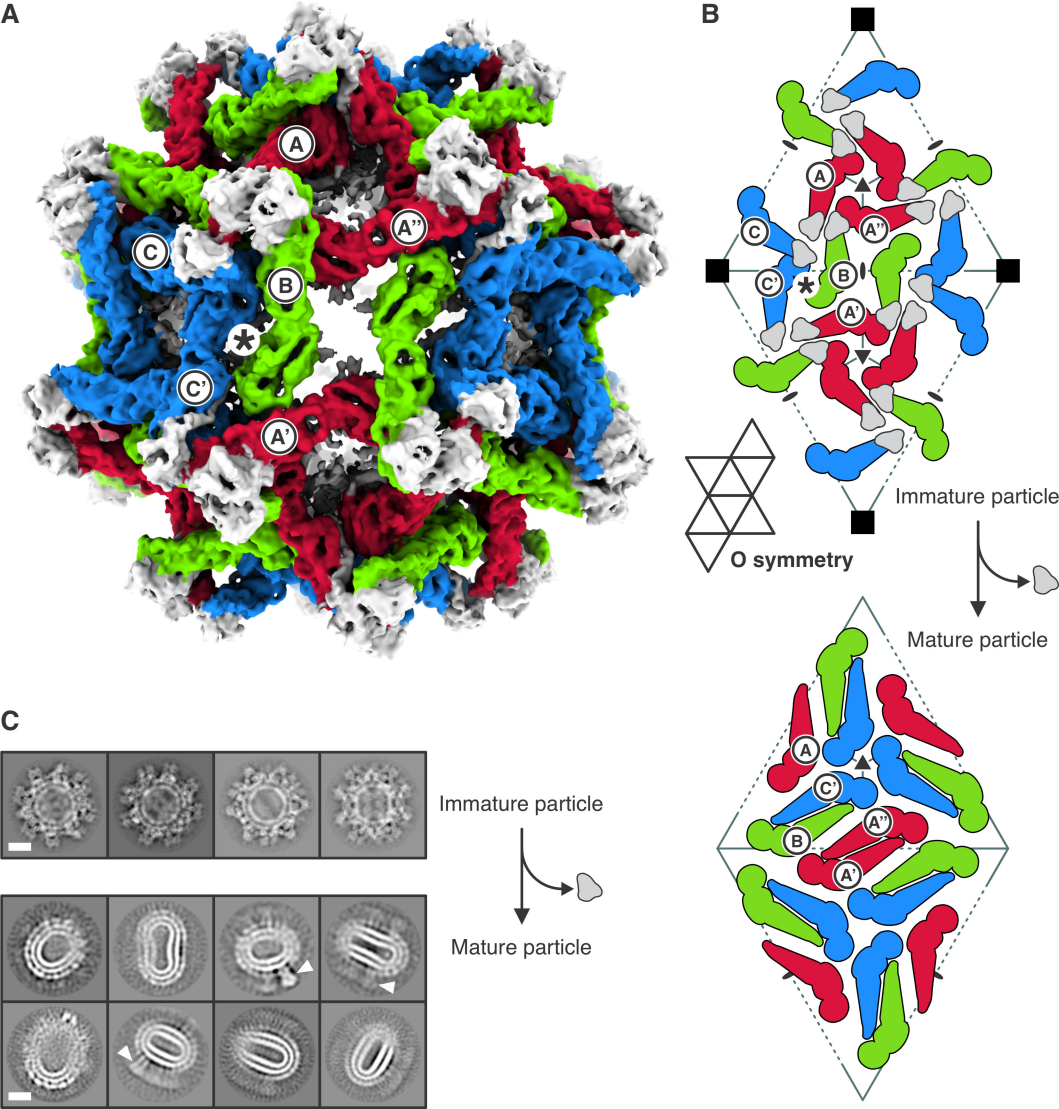
Immature and mature conformations of DENV2 smVLPs. (**A**) Structure of the octahedral particle with the asymmetric trimers in immature conformation. Protomers are colored red, green, and blue. prM of all protomers is shown in gray. Protomers of the reference asymmetric unit are labeled Ⓐ, Ⓑ, and Ⓒ. Protomers of neighboring asymmetric units are labeled with a prime or double prime, e.g., A′, and A″. Asterisk shows the position of a local twofold axis relating protomers B and C′. (**B**) Arrangement of prM-E protomers in the immature particle (top). Subunit labels and colors as in (**A**). Note that the stem-TM of E (not shown explicitly) will enter the membrane beneath the center of an adjacent asymmetric ABC trimer. Thus, the stem-TM of the E subunit labeled C′ will contact the stem-TM of prM on subunit B. In the transition to a mature particle, E subunits C′ and B dimerize, and their associated M fragments (stem-TM of prM after the pr fragment dissociates) remain together. Retention of octahedral symmetry during maturation (i.e., cleavage of pr-M and loss of pr) would yield the packing of M-E protomers shown in the bottom panel. The elongated, mature smVLPs show, however, that octahedral symmetry is not retained. We describe in the text the reasons to propose that the dimer pairing illustrated is present. (**C**) 2D class averages of immature (top) and mature (bottom) DENV2 smVLPs. White arrowheads indicate what appear to be E trimers in post-fusion conformation. The scale bar corresponds to 100 Å.

How does the subunit packing on the surface of an immature virus particle transform into the arrangement on the surface of a mature particle? The pattern of rearrangement proposed for BinJV (5) suggests that (i) the E subunits marked B and C in Fig. 4A rearrange into the BC dimer, with the C subunits clustered around the fivefold (fourfold in case of our smVLP), and (ii) one of the three E subunits related by the threefold axis (one of which is marked A) pair to form an AA′ dimer (across an icosahedral twofold). As the authors of that paper point out, this model requires a non-icosahedrally symmetric intermediate. But symmetry breaking would then require that the transition propagate asymmetrically from the proposed starting point. Moreover, broken symmetry seems hard to reconcile with the reversibility of the transition under *in vitro* conditions in which exposure of the furin site does not lead to its cleavage (19), as also pointed out by the authors of the SPOV structure (6). An alternative would be to pair the two A subunits related by an icosahedral twofold in the immature form (A′ and A″ in Fig. 4A and B), requiring those subunits to move and rotate substantially, but allowing retention of icosahedral symmetry throughout the transition and symmetrically related pairings across the entire particle. The SPOV paper illustrates this alternative in its supplementary movie 2 (6). In principle, it would be possible to rotate B and C′ in the opposite direction, so that B subunits cluster around the fivefold in the mature structure. Either alternative is compatible with retention of icosahedral symmetry. Subunits B and C′ are related to each other by a local twofold in both the immature and mature lattices (asterisks in Fig. 4A and B) and also in the octahedrally symmetric, immature smVLP (asterisk in Fig. 2A). Thus, BC′ pairing retains local twofold symmetry. Retention of this local twofold would also retain interactions in both the immature and mature lattices between the stem-TM of prM and the stem-TM of E, because the stem-TM of the E subunit marked B contacts the stem-TM of prM associated with the subunit marked C′, and vice versa (see caption of Fig. 4B).

The octahedral, immature smVLP could, in principle, retain octahedral symmetry as it transforms into a mature particle (see Fig. 4B and comment in the figure legend). The packing interactions seen in mature virions are apparently strong enough to destabilize a uniformly octahedral surface, which would require a fourfold clustering of domain III (the knob at the wide end of the green subunits in Fig. 4B), rather than the fivefold clustering in the icosahedral particle, and sharper curvature at the twofold (red-red dimer in Fig. 4B) than in the larger virion. Any icosahedral packing cannot, of course, cover the full surface, as it would require loss of six dimers together with about 17% of the bilayer. The ovoid mature smVLPs appear to have various local compromises, varying across a particle and between particles; those local packings might include instances of the one that would characterize the fully octahedral alternative.

The fusion properties of mature smVLPs of TBEV (13) and West Nile virus (WNV) (14) are indistinguishable from those of virions. Moreover, the kinetics of fusion by dengue smVLPs (all four serotypes) closely resemble those of WNV smVLPs (17). Cleavage of prM and release of the pr fragment must therefore allow E to rearrange as dimers on the smVLP surface into a packing that resembles, at least locally, the organization of E dimers on a virus particle. Otherwise, we would expect the kinetics and pH dependence of fusion to be different. Our mature dengue smVLPs are too irregular for a cryo-EM reconstruction (Fig. 4C), but studies of TBEV smVLPs, before the asymmetry of the immature trimer was known, suggested a *T* = 1 icosahedral structure (18). A similar interpretation has also been described for cryo-EM reconstructions of mature dengue type 2 smVLPs (16). How can we reconcile those observations with a 72-subunit immature precursor? We regard it as unlikely that subunits (and lipids) could be lost during a transition. The analysis of TBEV smVLPs was based on selected particles that were circular in outline, and they could therefore have been ovoid particles viewed near or along their major axis (18). The same might have been true of the dengue smVLPs examined by (16), in which particles were selected from a heterogeneous mixture of sizes. Clustering of BC dimers around a fivefold axis is a principal feature of subunit packing on the surface of mature virions, and it is plausible that a few, similar fivefold interactions would form as the smVLPs convert from immature to mature (see Fig. 4B and description in caption). Imposition of icosahedral symmetry in calculating the reconstruction would inevitably have yielded a symmetric map, probably with low-resolution features of the local fivefold interactions we suggest will form.

VLPs are candidates for the development of safe, flavivirus vaccines (16, 20, 21). Depending on the virus, expression strategies, and production conditions, the particles are probably a mixture of small and full-size VLPs. Epitope exposure is likely to be somewhat different on the mature versions of the two particle sizes, and vaccine trials with such particles will need to pay attention to the mixture present in the immunogen.

## MATERIALS AND METHODS

### Purification of DENV2 VLPs

We produced DV2 VLPs as described in refs (14, 17) with some modifications. HEK 293T cells stably expressing the DENV2 prME gene were grown as adherent cultures by passaging them in Dulbecco's modified Eagle medium (DMEM) High Glucose medium supplemented with 10% fetal bovine serum (FBS), 1% PenStrep, and 150 µg/mL puromycin. On day 1, cells were transferred into suspension culture at 1 million cells/mL in 293 Freestyle media and incubated for 4 days at 28°C. Cells were then removed by centrifugation, and VLPs were precipitated from the supernatant by adding PEG-8000 and NaCl to final concentrations of 8% (wt/vol) and 0.5 M, respectively, and incubating overnight at 4°C. The mixture was then centrifuged at 3,500 × *g*, and the pellet was resuspended in 20 mM HEPES (pH 7.5) and 150 mM NaCl for 20 min at 4°C. Centrifugation at 5,000 × *g* for 20 min clarified the solution, and the supernatant was then applied to a 5/10/15/20/60% (wt/vol) sucrose step gradient and centrifuged for 20 h in an SW41 rotor at 38,000 rpm. The 20% (wt/vol) sucrose fraction was collected and buffer exchanged to 20 mM HEPES (pH 7.5), 150 mM NaCl with an Amicon 100 kDa cutoff concentrator. VLPs were concentrated to 1 mg/mL protein, as estimated by SDS-PAGE and Coomassie staining.

### Cryo-EM grid preparation and data collection

Samples were vitrified using a Cryoplunge 3 robot. We applied 3.5 µL purified VLPs (1 mg/mL total protein concentration) to copper mesh holey carbon grids (C-flat 1.2 µm diameter holes with 1.3 µm spacing and a support thickness of ~20 nm), blotted for 4 s, and flash-frozen in liquid nitrogen-cooled liquid ethane. The humidity was kept at >90% during freezing. We collected movie image stacks with a Gatan K2 Summit direct detector on a TF30 Polara electron microscope, operated at 300 kV accelerating voltage, using SerialEM (22). The calibrated magnification at the physical pixel size was 40,650, and the defocus range was between −1.0 and −3.0 µm, resulting in a physical pixel size of 1.23 Å at the image plane. We collected three datasets in super-resolution counting mode with an electron dose rate of 8 electrons per physical pixel area per second and an exposure time of 200 ms per frame (40 frames per movie), resulting in a total exposure time of 8 s and a total electron dose of 42.3 electrons per Å^2^ per movie (1.06 electrons per Å^2^ per frame).

### Cryo-EM data processing

Movie frames were aligned (5 × 5 patch), averaged, and binned two times to a physical pixel size of 1.23Å with MotionCor2 (23). Initial contrast transfer function (CTF) parameters were determined with CTFFIND4 (24) from the summed micrographs. We first calculated a low-resolution smVLP map with octahedral symmetry imposed from a subset of manually picked particles using Relion (25), which was then used to generate reference projections with EMAN2 (26) for particle picking with Gautomatch (Fig. S3A). After local defocus estimation with Gctf (27) and 2D classification in Relion (25), we retained 38,934 particle images with a box size of 512 × 512 pixels (Fig. S3B). Particles were aligned with cisTEM (28) (refine3d version 1.01, reconstruct3d version 1.02); we imposed octahedral symmetry (O) and limited the alignment resolution to 8 Å. After local movie frame alignment and determination of optimal weighting factors for frame summation (relion_motion_refine), and estimation of CTF aberrations (relion_ctf_refine) (29), the nominal resolution of the octahedral smVLP reconstruction was 6.5 Å as determined by the Fourier shell correlation (FSC) between half maps (Fig. S4A; Table S1), similar to what was previously reported for cryo-EM reconstructions of SPOV (6) and BinJV (5) immature virus particles. For a local reconstruction, focusing on a single smVLP asymmetric unit, we symmetry expanded the particle stack, signal-subtracted the 6.5 Å-resolution density of the smVLP (except for the single asymmetric unit in question), and extracted a 256 × 256-pixel particle stack containing 934,416 images, each centered at one of the symmetry-related positions. After alignment by classification without alignment (30), the images and associated metadata were imported into CryoSPARC (31). A 3D classification into 10 classes without alignment was applied to the image stack; particles that partitioned into lower resolution classes were discarded. This step led to a stack of 706,684 particles and a reconstruction at 4.49 Å resolution. Another round of 3D classification into four classes led to a stack of 403,357 particles, from which a final local refinement was performed with adaptive marginalization and non-uniform refinement (32), limiting the rotational search to 20° and the shifts to 10 Å. The final reconstruction had a nominal resolution of 4.24 Å (Fig. S4B; Table S1). Data analysis, modeling, and refinement software were curated by SBGrid (33).

### Structure modeling and refinement

We used AlphaFold 2 (34) to obtain a model of a prM-E protomer, for which we input the sequences for pr, M, and E, respectively, as individual chains. From the resulting model, we placed the following domains into the corresponding densities of the three protomers in the 4.3 Å-resolution map of the local reconstruction: pr (residues 1–91), M (residues 110–166), E DI (residues 1–51, 131–191, 271–298), E DII (residues 52–130, 192–270), and E DIII (residues 299–393). Part of the E-protein stem and C-terminal domain (residues 394–449 out of residues 394–495) was taken from the model of the immature SPOV particle (PDB-ID 6ZQJ) and mutated to the DENV2 sequence (Data set S1 in the supplemental material), because AlphaFold 2 modeled the structure in the mature conformation, and therefore, this part did not fit the density of the immature particle. Domains were rigid-body fit and adjusted to the density with phenix.real_space refine using morphing (35). We then used RosettaCM (36) to fix the connections between the fitted domains. 3-mer and 9-mer fragment libraries were obtained from the Robetta server (http://robetta.bakerlab.org). We incorporated secondary structure restraint terms and the fit to the density map in the RosettaCM scoring function (37). We used the program O (38) to manually model the linker sequences connecting the pr and M domains (residues 92–109). The complete model consisting of prM-E protomers A, B, and C was refined with phenix.real_space (35). To obtain a model of the octahedral particle, we took the refined model of the asymmetric unit, rigid-body fit it into the 6.5 A-resolution octahedral map, and symmetry-expanded the structure to generate the full particle. The FSC between the refined models and the cryo-EM maps are shown in Fig. S4C and D. Map and model statistics are summarized in Table S1.

### Sequence alignments

Flavivirus sequences (Data set S1 to S3 in the supplemental material) were retrieved from the NCBI Viral Genomes Resource (39) with the following accession codes: dengue virus type 2 (DENV2), NP_056776.2; dengue virus type 1 (DENV1), NP_059433.1; dengue virus type 3 (DENV3), YP_001621843.1; dengue virus type 4 (DENV4), NP_073286.1; Japanese encephalitis virus (JEV), NP_059434.1; West Nile virus (WNV), YP_001527877.1; St. Louis encephalitis virus (SLEV), YP_001008348.1; Spondweni virus (SPOV), YP_009222008.1; Zika virus (ZIKV), YP_002790881.1; Powassan virus (POWV), NP_620099.1; tick-borne encephalitis virus (TBEV), NP_043135.1; yellow fever virus (YFV), NP_041726.1. Sequences were aligned with MAFFT (40), and multiple sequence alignments were printed and annotated with ESPript (41).

## Data Availability

Cryo-EM maps and refined models have been deposited in the Electron Microscopy Data Bank and Protein Data Bank, respectively, with accession identifiers EMD-47082 and PDB-ID 9DOF for the local reconstruction of the asymmetric unit, and EMD-47083 and PDB-ID 9DOG for the full octahedral smVLP.

## SUPPLEMENTAL MATERIAL

Supplemental material jvi.01809-24-s0001.pdf Figures S1 to S5, Table S1, and Data Sets S1 to S5.

ASM does not own the copyrights to Supplemental Material that may be linked to, or accessed through, an article. The authors have granted ASM a non-exclusive, world-wide license to publish the Supplemental Material files. Please contact the corresponding author directly for reuse.

## References

[1] Kostyuchenko VA, Zhang Q, Tan JL, Ng TS, Lok SM. 2013. Immature and mature dengue serotype 1 virus structures provide insight into the maturation process. J Virol 87:7700–7707. doi:10.1128/JVI.00197-13 23637416 PMC3700294

[2] Prasad VM, Miller AS, Klose T, Sirohi D, Buda G, Jiang W, Kuhn RJ, Rossmann MG. 2017. Structure of the immature Zika virus at 9 Å resolution. Nat Struct Mol Biol 24:184–186. doi:10.1038/nsmb.3352 28067914 PMC5296287

[3] Yu IM, Holdaway HA, Chipman PR, Kuhn RJ, Rossmann MG, Chen J. 2009. Association of the pr peptides with dengue virus at acidic pH blocks membrane fusion. J Virol 83:12101–12107. doi:10.1128/JVI.01637-09 19759134 PMC2786737

[4] Zhang Y, Kaufmann B, Chipman PR, Kuhn RJ, Rossmann MG. 2007. Structure of immature West Nile virus. J Virol 81:6141–6145. doi:10.1128/JVI.00037-07 17376919 PMC1900247

[5] Newton ND, Hardy JM, Modhiran N, Hugo LE, Amarilla AA, Bibby S, Venugopal H, Harrison JJ, Traves RJ, Hall RA, Hobson-Peters J, Coulibaly F, Watterson D. 2021. The structure of an infectious immature flavivirus redefines viral architecture and maturation. Sci Adv 7. doi:10.1126/sciadv.abe4507 PMC8121421 33990320

[6] Renner M, Dejnirattisai W, Carrique L, Martin IS, Karia D, Ilca SL, Ho SF, Kotecha A, Keown JR, Mongkolsapaya J, Screaton GR, Grimes JM. 2021. Flavivirus maturation leads to the formation of an occupied lipid pocket in the surface glycoproteins. Nat Commun 12:1238. doi:10.1038/s41467-021-21505-9 33623019 PMC7902656

[7] Kuhn RJ, Zhang W, Rossmann MG, Pletnev SV, Corver J, Lenches E, Jones CT, Mukhopadhyay S, Chipman PR, Strauss EG, Baker TS, Strauss JH. 2002. Structure of dengue virus: implications for flavivirus organization, maturation, and fusion. Cell 108:717–725. doi:10.1016/s0092-8674(02)00660-8 11893341 PMC4152842

[8] Zhang X, Ge P, Yu X, Brannan JM, Bi G, Zhang Q, Schein S, Zhou ZH. 2013. Cryo-EM structure of the mature dengue virus at 3.5-Å resolution. Nat Struct Mol Biol 20:105–110. doi:10.1038/nsmb.2463 23241927 PMC3593067

[9] Modis Y, Ogata S, Clements D, Harrison SC. 2004. Structure of the dengue virus envelope protein after membrane fusion. Nature New Biol 427:313–319. doi:10.1038/nature02165 14737159

[10] Anastasina M, Füzik T, Domanska A, Pulkkinen LIA, Šmerdová L, Formanová PP, Straková P, Nováček J, Růžek D, Plevka P, Butcher SJ. 2024. The structure of immature tick-borne encephalitis virus supports the collapse model of flavivirus maturation. Sci Adv 10:eadl1888. doi:10.1126/sciadv.adl1888 38959313 PMC11221509

[11] Majowicz SA, Narayanan A, Moustafa IM, Bator CM, Hafenstein SL, Jose J. 2023. Zika virus M protein latches and locks the E protein from transitioning to an immature state after prM cleavage. NPJ Viruses 1:4. doi:10.1038/s44298-023-00004-2 PMC11721464 40295858

[12] Allison SL, Mandl CW, Kunz C, Heinz FX. 1994. Expression of cloned envelope protein genes from the flavivirus tick-borne encephalitis virus in mammalian cells and random mutagenesis by PCR. Virus Genes 8:187–198. doi:10.1007/BF01703077 7975266

[13] Schalich J, Allison SL, Stiasny K, Mandl CW, Kunz C, Heinz FX. 1996. Recombinant subviral particles from tick-borne encephalitis virus are fusogenic and provide a model system for studying flavivirus envelope glycoprotein functions. J Virol 70:4549–4557. doi:10.1128/JVI.70.7.4549-4557.1996 8676481 PMC190391

[14] Chao LH, Klein DE, Schmidt AG, Peña JM, Harrison SC. 2014. Sequential conformational rearrangements in flavivirus membrane fusion. Elife 3:e04389. doi:10.7554/eLife.04389 25479384 PMC4293572

[15] Allison SL, Tao YJ, O’Riordain G, Mandl CW, Harrison SC, Heinz FX. 2003. Two distinct size classes of immature and mature subviral particles from tick-borne encephalitis virus. J Virol 77:11357–11366. doi:10.1128/jvi.77.21.11357-11366.2003 14557621 PMC229348

[16] Shen W-F, Galula JU, Liu J-H, Liao M-Y, Huang C-H, Wang Y-C, Wu H-C, Liang J-J, Lin Y-L, Whitney MT, Chang G-JJ, Chen S-R, Wu S-R, Chao D-Y. 2018. Epitope resurfacing on dengue virus-like particle vaccine preparation to induce broad neutralizing antibody. Elife 7. doi:10.7554/eLife.38970 PMC6234032 30334522

[17] Chao LH, Jang J, Johnson A, Nguyen A, Gray NS, Yang PL, Harrison SC. 2018. How small-molecule inhibitors of dengue-virus infection interfere with viral membrane fusion. Elife 7:e36461. doi:10.7554/eLife.36461 29999491 PMC6056230

[18] Ferlenghi I, Clarke M, Ruttan T, Allison SL, Schalich J, Heinz FX, Harrison SC, Rey FA, Fuller SD. 2001. Molecular organization of a recombinant subviral particle from tick-borne encephalitis virus. Mol Cell 7:593–602. doi:10.1016/s1097-2765(01)00206-4 11463384

[19] Yu IM, Zhang W, Holdaway HA, Li L, Kostyuchenko VA, Chipman PR, Kuhn RJ, Rossmann MG, Chen J. 2008. Structure of the immature dengue virus at low pH primes proteolytic maturation. Science 319:1834–1837. doi:10.1126/science.1153264 18369148

[20] Thoresen D, Matsuda K, Urakami A, Ngwe Tun MM, Nomura T, Moi ML, Watanabe Y, Ishikawa M, Hau TTT, Yamamoto H, Suzaki Y, Ami Y, Smith JF, Matano T, Morita K, Akahata W. 2024. A tetravalent dengue virus-like particle vaccine induces high levels of neutralizing antibodies and reduces dengue replication in non-human primates. J Virol 98:e0023924. doi:10.1128/jvi.00239-24 38647327 PMC11092354

[21] Cuevas-Juárez E, Pando-Robles V, Palomares LA. 2021. Flavivirus vaccines: virus-like particles and single-round infectious particles as promising alternatives. Vaccine (Auckl) 39:6990–7000. doi:10.1016/j.vaccine.2021.10.049 34753613

[22] Mastronarde DN. 2005. Automated electron microscope tomography using robust prediction of specimen movements. J Struct Biol 152:36–51. doi:10.1016/j.jsb.2005.07.007 16182563

[23] Zheng SQ, Palovcak E, Armache JP, Verba KA, Cheng Y, Agard DA. 2017. MotionCor2: anisotropic correction of beam-induced motion for improved cryo-electron microscopy. Nat Methods 14:331–332. doi:10.1038/nmeth.4193 28250466 PMC5494038

[24] Rohou A, Grigorieff N. 2015. CTFFIND4: fast and accurate defocus estimation from electron micrographs. J Struct Biol 192:216–221. doi:10.1016/j.jsb.2015.08.008 26278980 PMC6760662

[25] Scheres SHW. 2012. RELION: implementation of a Bayesian approach to cryo-EM structure determination. J Struct Biol 180:519–530. doi:10.1016/j.jsb.2012.09.006 23000701 PMC3690530

[26] Tang G, Peng L, Baldwin PR, Mann DS, Jiang W, Rees I, Ludtke SJ. 2007. EMAN2: an extensible image processing suite for electron microscopy. J Struct Biol 157:38–46. doi:10.1016/j.jsb.2006.05.009 16859925

[27] Zhang K. 2016. Gctf: Real-time CTF determination and correction. J Struct Biol 193:1–12. doi:10.1016/j.jsb.2015.11.003 26592709 PMC4711343

[28] Grant T, Rohou A, Grigorieff N. 2018. cisTEM, user-friendly software for single-particle image processing. Elife 7:e35383. doi:10.7554/eLife.35383 29513216 PMC5854467

[29] Scheres SH. 2014. Beam-induced motion correction for sub-megadalton cryo-EM particles. Elife 3:e03665. doi:10.7554/eLife.03665 25122622 PMC4130160

[30] Jenni S, Li Z, Wang Y, Bessey T, Salgado EN, Schmidt AG, Greenberg HB, Jiang B, Harrison SC. 2022. Rotavirus VP4 epitope of a broadly neutralizing human antibody defined by its structure bound with an attenuated-strain virion. J Virol 96:e0062722. doi:10.1128/jvi.00627-22 35924923 PMC9400500

[31] Punjani A, Rubinstein JL, Fleet DJ, Brubaker MA. 2017. cryoSPARC: algorithms for rapid unsupervised cryo-EM structure determination. Nat Methods 14:290–296. doi:10.1038/nmeth.4169 28165473

[32] Punjani A, Zhang H, Fleet DJ. 2020. Non-uniform refinement: adaptive regularization improves single-particle cryo-EM reconstruction. Nat Methods 17:1214–1221. doi:10.1038/s41592-020-00990-8 33257830

[33] Morin A, Eisenbraun B, Key J, Sanschagrin PC, Timony MA, Ottaviano M, Sliz P. 2013. Collaboration gets the most out of software. Elife 2:e01456. doi:10.7554/eLife.01456 24040512 PMC3771563

[34] Jumper J, Evans R, Pritzel A, Green T, Figurnov M, Ronneberger O, Tunyasuvunakool K, Bates R, Žídek A, Potapenko A, et al.. 2021. Highly accurate protein structure prediction with AlphaFold. Nature New Biol 596:583–589. doi:10.1038/s41586-021-03819-2 PMC8371605 34265844

[35] Afonine PV, Poon BK, Read RJ, Sobolev OV, Terwilliger TC, Urzhumtsev A, Adams PD. 2018. Real-space refinement in PHENIX for cryo-EM and crystallography. Acta Crystallogr D Struct Biol 74:531–544. doi:10.1107/S2059798318006551 29872004 PMC6096492

[36] Song Y, DiMaio F, Wang RY-R, Kim D, Miles C, Brunette T, Thompson J, Baker D. 2013. High-resolution comparative modeling with RosettaCM. Structure 21:1735–1742. doi:10.1016/j.str.2013.08.005 24035711 PMC3811137

[37] Wang RY-R, Kudryashev M, Li X, Egelman EH, Basler M, Cheng Y, Baker D, DiMaio F. 2015. De novo protein structure determination from near-atomic-resolution cryo-EM maps. Nat Methods 12:335–338. doi:10.1038/nmeth.3287 25707029 PMC4435692

[38] Jones TA, Zou JY, Cowan SW, Kjeldgaard M. 1991. Improved methods for building protein models in electron density maps and the location of errors in these models. Acta Crystallogr A 47 (Pt 2):110–119. doi:10.1107/s0108767390010224 2025413

[39] Brister JR, Ako-adjei D, Bao Y, Blinkova O. 2015. NCBI viral genomes resource. Nucleic Acids Res 43:D571–D577. doi:10.1093/nar/gku1207 25428358 PMC4383986

[40] Katoh K, Misawa K, Kuma K, Miyata T. 2002. MAFFT: a novel method for rapid multiple sequence alignment based on fast Fourier transform. Nucleic Acids Res 30:3059–3066. doi:10.1093/nar/gkf436 12136088 PMC135756

[41] Robert X, Gouet P. 2014. Deciphering key features in protein structures with the new ENDscript server. Nucleic Acids Res 42:W320–4. doi:10.1093/nar/gku316 24753421 PMC4086106

[42] Pettersen EF, Goddard TD, Huang CC, Meng EC, Couch GS, Croll TI, Morris JH, Ferrin TE. 2021. UCSF ChimeraX: structure visualization for researchers, educators, and developers. Protein Sci 30:70–82. doi:10.1002/pro.3943 32881101 PMC7737788

[43] Hunter JD. 2007. Matplotlib: a 2D graphics environment. Comput Sci Eng 9:90–95. doi:10.1109/MCSE.2007.55

