# Supplementary material for: Octahedral small virus-like particles of dengue virus type 2
Source: J Virol. 2024 Dec 31;99(2):e01809-24. doi: 10.1128/jvi.01809-24 (PMC11853069; doi:10.1128/jvi.01809-24)
Supplement: Supplemental material — Figures S1 to S5, Table S1, and Data Sets S1 to S5. [file jvi.01809-24-s0001.pdf]

Supplemental material

Octahedral small virus-like particles of dengue virus type 2

Adam Johnson[1,*], Martín Dodes Traian[1], Richard M. Walsh Jr.[1], Simon Jenni[1,†], Stephen C. Harrison[1,2,3,†]

[1]Department of Biological Chemistry and Molecular Pharmacology, Harvard Medical School, 250 Longwood Avenue, Boston, MA 02115, USA.
[2]Laboratory of Molecular Medicine, Boston Children's Hospital, Boston, MA 02115, USA
[3]Howard Hughes Medical Institute, Harvard Medical School, Boston, MA 02115, USA
* Current address: Vertex Pharmaceuticals, Boston, MA 02210, USA

[†]Correspondence: jenni@crystal.harvard.edu, harrison@crystal.harvard.edu

Phone: 617-432-5607 Fax: 617-432-5600

This PDF includes :

FIG S1 to S5

TABLE S1

Data set S1 to S3

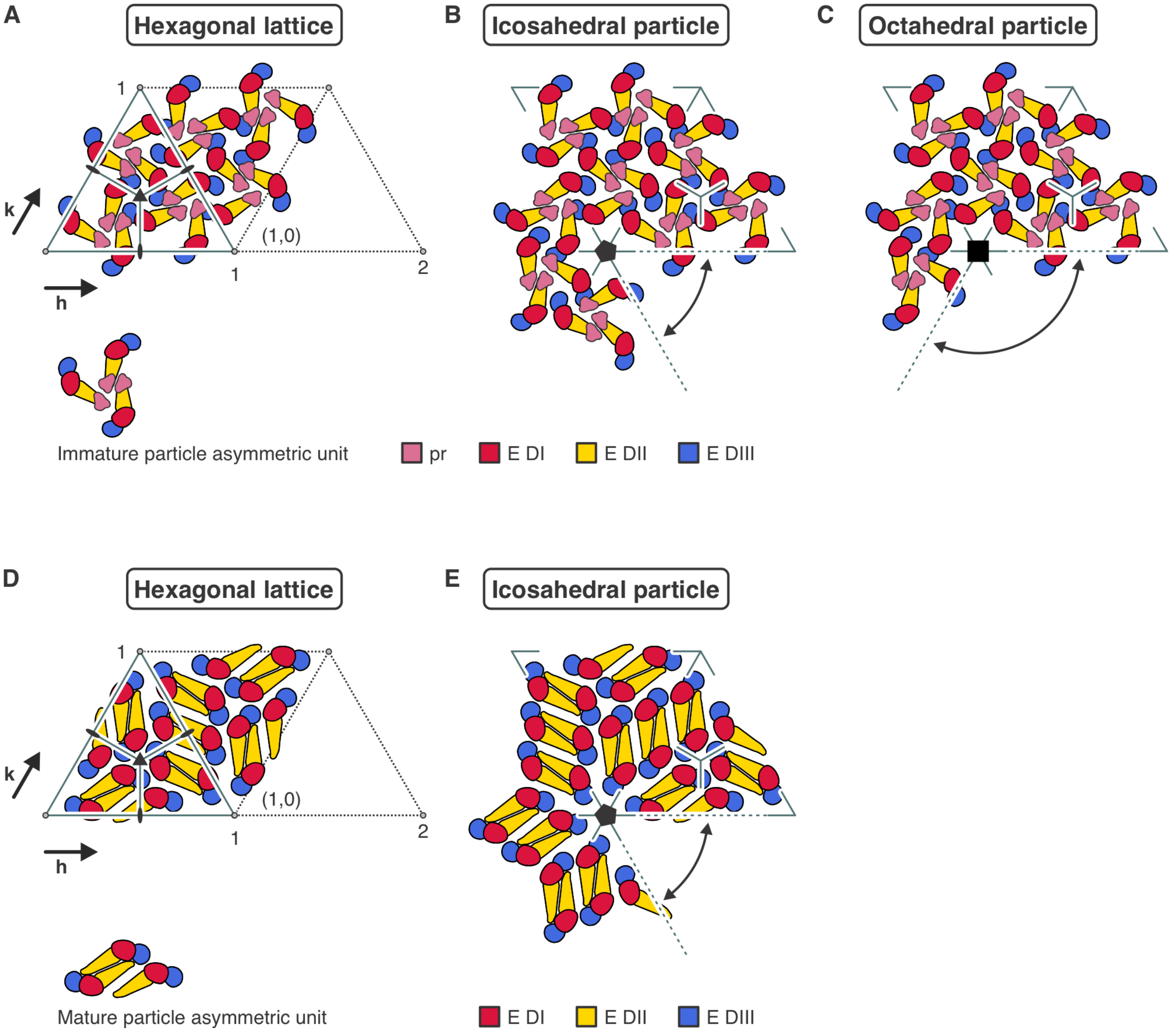

FIG S1 Schematic illustration of subunit packing in immature and mature flavivirus particles with a domain coloring scheme (domains I, II, III in red, yellow and blue, respectively). The folding of a hypothetical planar, hexagonal (p6) lattice follows the scheme of Caspar and Klug (44). ((A) The immature conformation of prM-E on a p6 lattice with an asymmetric trimer within each asymmetric unit. (B) Folding of triangular faces to generate the 5-fold axes of an icosahedral immature particle with one asymmetric trimer within the icosahedral asymmetric unit (T=1). Each sixfold axis of the p6 lattice becomes a fivefold axis of the icosahedral particle. (C) Folding of triangular faces to generate the 4-fold axes of an octahedral immature particle. Each sixfold axis of the p6 lattice becomes a fourfold axis of the octahedral particle. (D) The mature conformation of M-E on a p6 lattice with one and a half dimers within each asymmetric unit. (E) Folding of triangular faces to generate the 5-fold axes of an icosahedral mature particle.

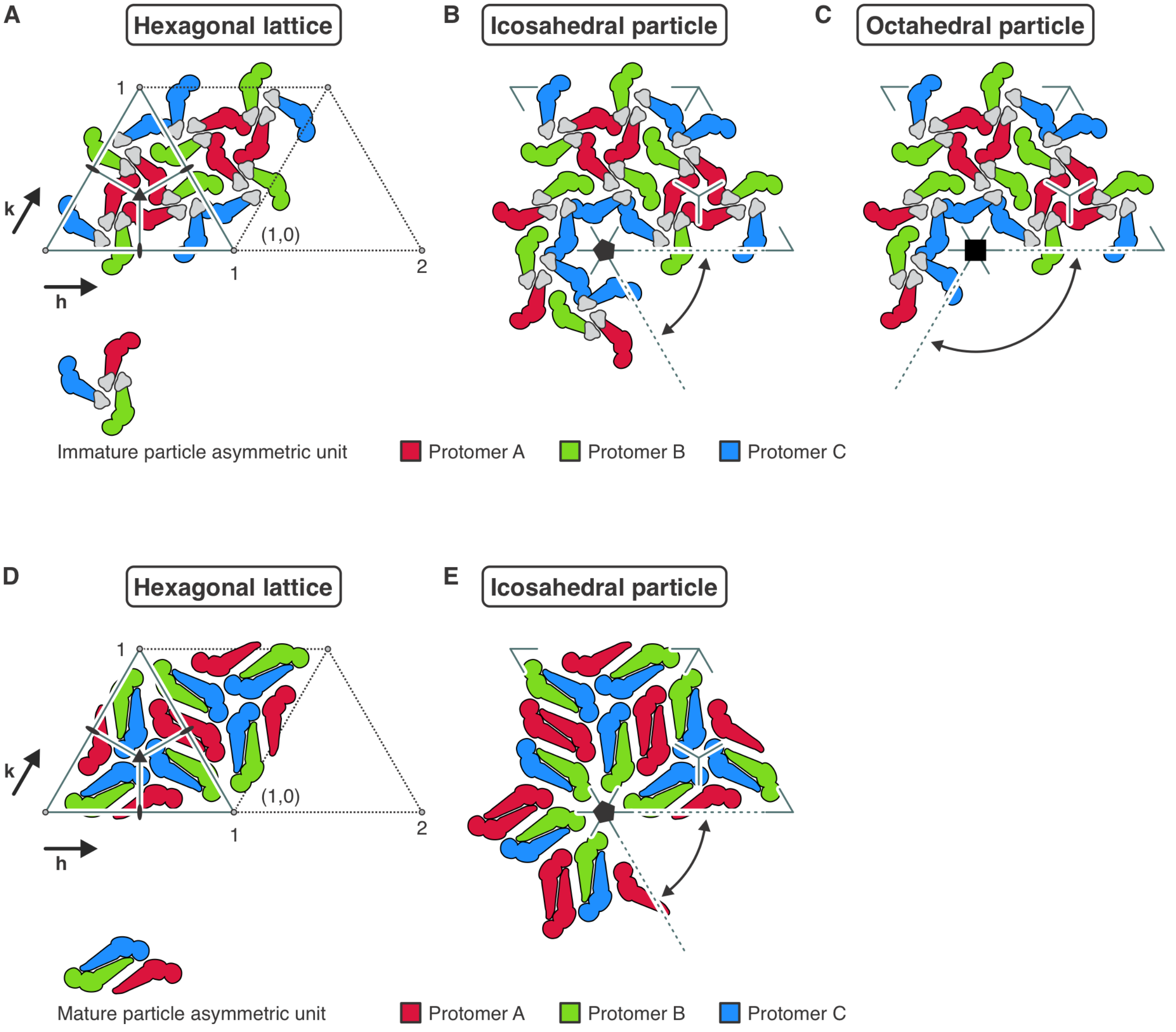

FIG S2 Schematic illustration of subunit packing in immature and mature flavivirus particles with a protomer coloring scheme. Protomers or each asymmetric unit are colored red, green, and blue. prM of all protomers is shown in gray. The folding of a hypothetical planar, hexagonal (p6) lattice follows the scheme of Caspar and Klug (1). (A) The immature conformation of prM-E on a p6 lattice with an asymmetric trimer within each asymmetric unit. (B) Folding of triangular faces to generate the 5-fold axes of an icosahedral immature particle. (C) Folding of triangular faces to generate the 4-fold axes of an octahedral immature particle. (D) The mature conformation of M-E on a pseudo hexagonal lattice with one and a half dimers within each asymmetric unit. (E) Folding of triangular faces to generate the 5-fold axes of an icosahedral mature particle.

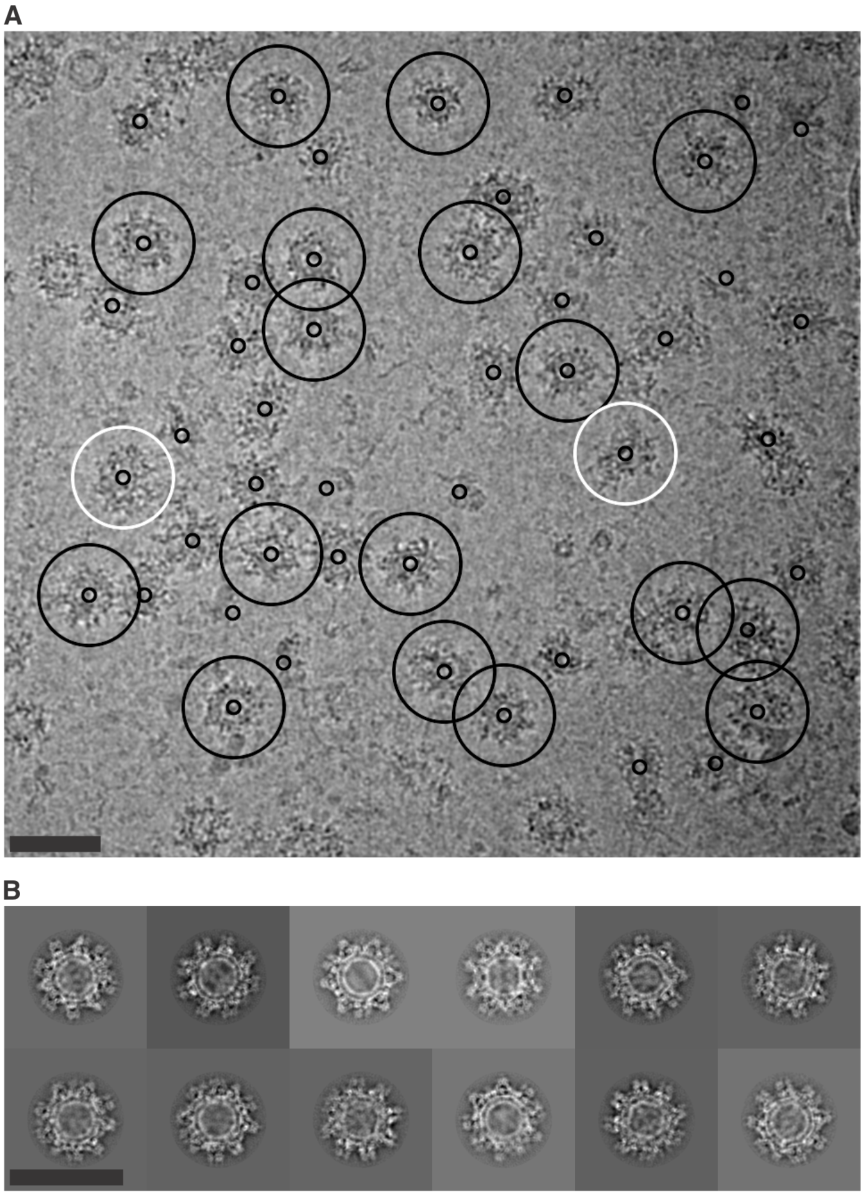

FIG S3 cryo-EM analysis of DENV2 smVLPs. (A) Low pass filtered micrograph. Small black circles: particles picked; large black circles: immature particles after 2D classification; large white circles: mature particles after 2D classification. The scale bar corresponds to 500 Å. (B) 2D class averages of immature DENV2 smVLPs. The scale bar corresponds to 500 Å.

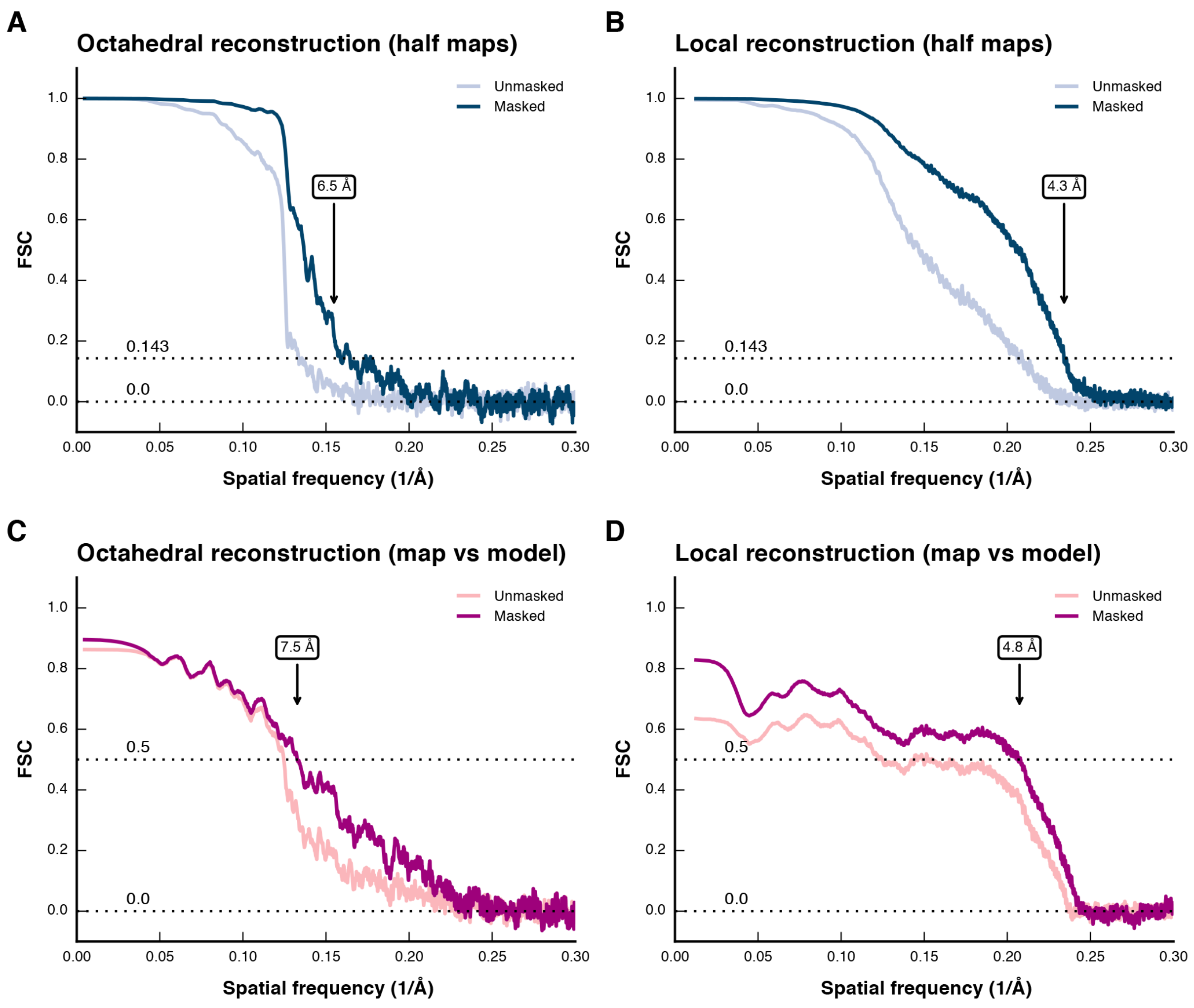

FIG S4 Fourier shell correlation (FSC) analysis. (A) and (B) FSC between half maps for the octahedral and local reconstructions, respectively. The nominal resolution at which the correlation drops below 0.143 is shown. Light blue, unmasked; dark blue, after applying a soft mask to the half maps. (C) and (D) FSC between the final map and the model for the octahedral and local reconstructions, respectively. The nominal resolution at which the correlation drops below 0.5 is shown. Light red, unmasked; dark red, after applying a soft mask to the final map.

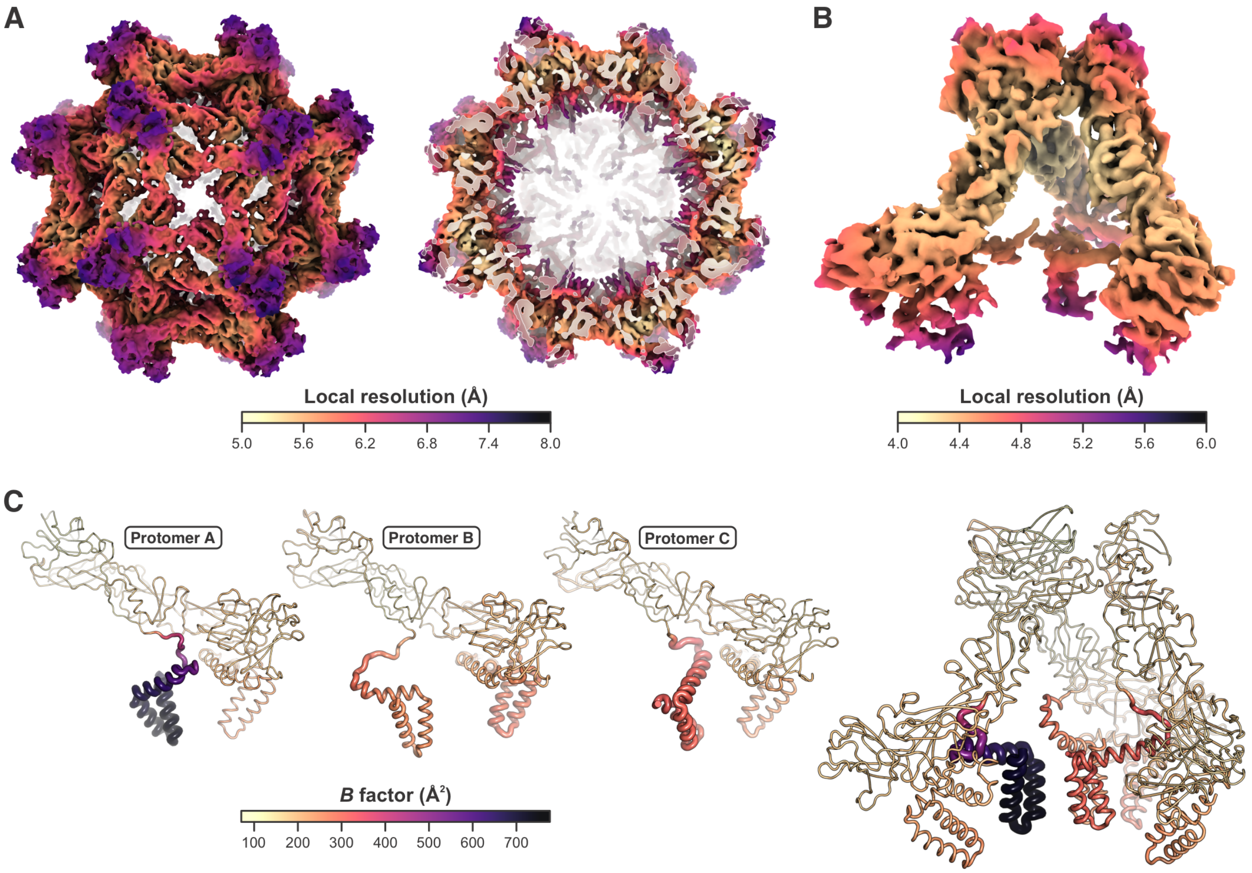

FIG S5 Local resolution and *B* factor analysis. (A) cryo-EM reconstruction of the full DENV2 smVLP with octahedral symmetry. (B) Local reconstruction of the asymmetric trimer. (C) *B* factors mapped on the refined structure of the asymmetric trimer. *B* factor values are only meaningful relative to other values in the structure, as they depend on the degree of sharpening that was applied to the cryo-EM reconstruction.

TABLE S1 Cryo-EM data collection and model statistics

	Octahedral small virus-like particles of dengue virus type 2	
**Data collection**		
Electron microscope	Polara	
Magnification	40,650	
Voltage (kV)	300	
Defocus range (μm) [a]	1.5–3.0	
Physical pixel size (Å)	1.23	
Number of movies	13,808	
**Octahedral reconstruction**		
Number of images	38,934	
Box size (pixels)	512	
Symmetry imposed	O	
Map resolution (Å) [b]	6.5	
**Local reconstruction**		
Number of images	403,357	
Box size (pixels)	256	
Symmetry imposed	C_1	
Map resolution (Å) [b]	4.3	
**Model statistics**	**Local reconstruction**	**Octahedral reconstruction**
EMD accession identifier	EMD-47082	EMD-47083
PDB accession identifier	9DOF	9DOG
Refinement resolution (Å)	4.3	6.5 (rigid body fitting only)
CC (mask)	0.67	0.71
Model composition		
Non-hydrogen atoms	15,354	
Protein residues	1,983	
B factors ($Å^2$)		
Min	65	
Max	782	
Mean	174	
R.m.s. deviations		
Bond lengths (Å)	0.005	
Bond angles (°)	0.948	
Validation		
MolProbity clash score	8.4	
Poor rotamers (%)	0.0	
Ramachandran plot		
Favored (%)	92.6	
Allowed (%)	7.1	
Disallowed (%)	0.3	

[a] Approximate range of underfocus.
[b] Resolution where FSC between masked half maps drops below 0.143.

Data set S1 Multiple sequence alignment of part of the flavivirus polyprotein covering prM-E. DENV2, dengue virus type 2; DENV1, dengue virus type 1; DENV3 dengue virus type 3; DENV4, dengue virus type 4; JEV, Japanese encephalitis virus; WNV, West Nile virus; SLEV, St. Louis encephalitis virus; SPOV, Spondweni virus; ZIKV, Zika virus; POWV, Powassan virus; TBEV, tick-borne encephalitis virus; YFV, yellow fever virus. Secondary structure elements are indicated above the sequences. Solid bars below the sequences are colored according to the following domains: pr, pink; M, brown; E DI, red; E DII, yellow; E DIII, blue; E stem and C-terminal domains, cyan. Proteolytic cleavage sites are indicated by double ovals. See Materials and Methods for NCBI Viral Genomes Resource accession codes.

Data set S2 Flavivirus prM protein multiple sequence alignment. DENV2, dengue virus type 2; DENV1, dengue virus type 1; DENV3 dengue virus type 3; DENV4, dengue virus type 4; JEV, Japanese encephalitis virus; WNV, West Nile virus; SLEV, St. Louis encephalitis virus; SPOV, Spondweni virus; ZIKV, Zika virus; POWV, Powassan virus; TBEV, tick-borne encephalitis virus; YFV, yellow fever virus. Secondary structure elements are indicated above the sequences. Solid bars below the sequences are colored according to the following domains: pr, pink; M, brown. The furin proteolytic cleavage sites is indicated by double ovals. See Materials and Methods for NCBI Viral Genomes Resource accession codes.

Data set S3 Flavivirus E protein multiple sequence alignment. DENV2, dengue virus type 2; DENV1, dengue virus type 1; DENV3 dengue virus type 3; DENV4, dengue virus type 4; JEV, Japanese encephalitis virus; WNV, West Nile virus; SLEV, St. Louis encephalitis virus; SPOV, Spondweni virus; ZIKV, Zika virus; POWV, Powassan virus; TBEV, tick-borne encephalitis virus; YFV, yellow fever virus. Secondary structure elements are indicated above the sequences. Solid bars below the sequences are colored according to the following domains: E DI, red; E DII, yellow; E DIII, blue; E stem and C-terminal domains, cyan. See Materials and Methods for NCBI Viral Genomes Resource accession codes.

Data set S4 prM protein inter-protomer contacts in the immature octahedral DENV2 particle (reported here) and the immature icosahedral SPOV particle (PDB-ID 6ZQW), respectively, mapped on the primary sequences. prM residues that are within a 5 Å distance to neighboring protomer residues are labeled with black (contacts to protomers of the same asymmetric unit) and gray (contacts to protomers of other asymmetric units) ovals. Contacts are mapped for each of the three protomers of the asymmetric units.

Data set S5 E protein inter-protomer contacts in the immature octahedral DENV2 particle (reported here) and the immature icosahedral SPOV particle (PDB-ID 6ZQW), respectively, mapped on the primary sequences. E residues that are within a 5 Å distance to neighboring protomer residues are labeled with black (contacts to protomers of the same asymmetric unit) and gray (contacts to protomers of other asymmetric units) ovals. Contacts are mapped for each of the three protomers of the asymmetric units.

Reference

1. Caspar DLD, Klug A. 1962. Physical principles in the construction of regular viruses. Cold Spring Harb Symp Quant Bio 27:1-24. https://doi.org/10/1101/SQB.1962.027.001.005

Data set 1

```
DSSP
DENV2          1  M.NNQ..RKKAKN.TPFNMLKRERNRV.STVQQLTKRFSLGMLQGRGPLKLFMALVAFLRFLTI...PPT
DENV1          1  M.NNQ..RKKTGR.PSFNMLKRARNRV.STVSQLAKRFSKGLLSGQGPMKLVMAFIAFLRFLAI...PPT
DENV3          1  M.NNQ..RKKTGK.PSINMLKRVRNRV.STGSQLAKRFSKGLLNGQGPMKLVMAFIAFLRFLAI...PPT
DENV4          1  M..NQ..RKKVVR.PPFNMLKRERNRV.STPQGLVKRFSTGLFSGKGPLRMVLAFITFLRVLSI...PPT
JEV            1  MTKKP..GGPGKN.RAINMLKRGLPRV.FPLVGV.KRVVMSLLDGRGPVRFVLALITFFKFTAL...APT
WNV            1  MSKKP..GGPGKS.RAVNMLKRGMPRV.LSLIGL.KRAMLSLIDGKGPIRFVLALLAFFRFTAI...APT
SLEV           1  MSKKP..GKPGRN.RVVNMLKRGVSRV.NPLTGL.KRILGSLLDGRGPVRFILAILTFFRFTAL...QPT
SPOV           1  M.KNP..KRAGSS.RLVNMLRRGAARVIPPGGGL.KRLPVGLLLGRGPIKMILAILAFLRFTAI...KPS
ZIKV           1  M.KNP..KEEIRRIRIVNMLKRGVARV.NPLGGL.KRLPAGLLLGHGPIRMVLAILAFLRFTAI...KPS
POWV           1  MMTTS..KGKGGG.PPRRKLKVTANKSRPATSPM.............PKGFVLSRMLGILWHAVTGTARP
TBEV           1  MVKKAILKGKGGG.PPRRVSKETATKTRQPRVQM.............PNGLVLMRMMGILWHAVAGTARN
YFV            1  M.SGR..KAQGKT.LGVNMVRRGVRSL...SNKI.KQKTKQIGNRPGPSRGVQGFIFFFLFNILTGKKIT
consensus>50      M...............nmlkr...rv......l.kr....l..g.gP...vla...f..f.ai....p.

DSSP
DENV2         63  AGILKRWGTIKKSKAINVLRGFRKEIGRMLNILNRRRRS.............AGMIIMLIPTVMAFHLTT
DENV1         63  AGILARWGSFKKNGAIKVLRGFKKEISNMLNIMNRRKRS.............VTMLLMLLPTALAFHLTT
DENV3         63  AGVLARWGTFKKSGAIKVLKGFKKEISNMLSIINQRKKT.............SLCLMMILPAALAFHLTS
DENV4         62  AGILKRWGQLKKNKAIKILIGFRKEIGRMLNILNGRKRS.............TITLLCLIPTVMAFSLST
JEV           63  KALLGRWKAVEKSVAMKHLTSFKRELGTLIDAVNKRGRKQNKRGGNEGSIMWLASLAVVIAYAGAMKLSN
WNV           63  RAVLDRWRGVNKQTAMKHLLSFKKELGTLTSAINRRSSKQKKRGGKTG....IAVMIGLIASVGAVTLSN
SLEV          63  EALKRRWRAVDKRTALKHLNGFKRDLGSMLDTINRRPSK..KRGGTRS....LLGLAALIGLASSLQLST
SPOV          63  TGLINRWGKVGKKEAIKILTKFKADVGTMLRIINNRKTK..KRGVETG....IVFLALLVSI.VAVEVTK
ZIKV          63  LGLINRWGSVGKKEAMEIIKKFKKDLAAMLRIINARKER.KRRGADTS....IGIIGLLLTTAMAAEITR
POWV          55  PVLKMFWKTVPLRQAEAVLKKIKRVIGNLMQSLHMRGRR..RSGVDWT..WIFLTMALMTMAMATTIHRD
TBEV          57  PVLKAFWNSVPLKQATAALRKIKRTVSALMVGLQKRGKR..RSATDWM..SWLLVITLLGMTLAATVRKE
YFV           63  AHLKRLWKMLDPRQGLAVLRKVKRVVASLMRGLSSRKRR..SHDVLTV...QFLILGMLLMT.GGVTLVR
consensus>50      .....rW....k..a...l..fk.e...$l...n.R.................l..l.....a.....

DSSP
DENV2        120  RNGEPHMIVSRQEKGKSLLFKTEDGVNMCTLMAMDLGELCEDTITYKCPLLR.QNEPEDIDCWC.NSTST
DENV1        120  RGGEPHMIVSKQERGKSLLFKTSAGVNMCTLIAMDLGELCEDTMTYKCPRIT.ETEPDDVDCWC.NATET
DENV3        120  RDGEPRMIVGKNERGKSLLFKTASGINMCTLIAMDLGEMCDDTVTYKCPHIT.EVEPEDIDCWC.NLTST
DENV4        119  RDGEPLMIVAKHERGRPLLFKTTEGINKCTLIAMDLGEMCEDTVTYKCPLLV.NTEPEDIDCWC.NLTST
JEV          133  FQGKLLMTINNTDIADVIVIPTSKGENRCWVRAIDVGYMCEDTITYECPKLTMGNDPEDVDCWC.DNQEV
WNV          129  FQGKVMMTVNATDVTDVITIPTAAGKNLCIVRAMDVGYMCDDTITYECPVLSAGNDPEDIDCWC.TKSAV
SLEV         127  YQGKVLMSINKTDAQSAINIPSANGANTCIVRALDVGVMCKDDITYLCPVLSAGNDPEDIDCWC.DVEEV
SPOV         126  KGDTYYMFADKKDAGKVVTFETESGPNRCSIQAMDIGHMCPATMSYECPVLEPQYEPEDVDCWC.NSTAA
ZIKV         128  RGSAYYMYLDRSDAGKAISFATTLGVNKCHVQIMDLGHMCDATMSYECPMLDEGVEPDDVDCWC.NTTST
POWV         121  REGYMVMRASGRDAASQVRVQN....GTCVILATDMGEWCEDSITYSCVTIDQEEEPVDVDCFCRGVDRV
TBEV         123  RDGSTVIRAEGKDAATQVRVEN....GTCVILATDMGSWCDDSLSYECVTIDQGEEPVDVDCFCRNVDGV
YFV          127  KNRWLLLNVTSEDLGKTFSV....GTGNCTTNILEAKYWCPDSMEYNCPNLSPREEPDDIDCWCYGVENV
consensus>50      .dg...m.....#..........g.n.C...am#.g.mCed...Y.Cp......#PeD!DCwC.n....

DSSP
DENV2        188  WVTYGTCTT..MGEHRREKRSVALVPHVGMGLETRTETWMSSEGAWKHVQRIETWILRHPGFTMMAAILA
DENV1        188  WVTYGTCSQ..TGEHRRDKRSVALAPHVGLGLETRTETWMSSEGAWKQIQKVETWALRHPGFTVIALFLA
DENV3        188  WVTYGTCNQ..AGEHRRDKRSVALAPHVGMGLDTRTQTWMSAEGAWRQVEKVETWALRHPGFTILALFLA
DENV4        187  WVMYGTCTQ..SGERRREKRSVALTPHSGMGLETRAETWMSSEGAWKHAQRVESWILRNPGFALLAGFMA
JEV          202  YVQYGRCTR..TRHSKRSRRSVSVQTHGESSLVNKKEAWLDSTKATRYLMKTENWIIRNPGYAFLAATLG
WNV          198  YVRYGRCTK..TRHSRRSRRSLTVQTHGESTLANKKGAWLDSTKATRYLVKTESWILRNPGYALVAAVIG
SLEV         196  WVHYGRCTR..MGHSRRSRRSISVQHHGDSTLATKNTPWLDTVKTTKYLTKVENWVLRNPGYALVALAIG
SPOV         195  WIVYGTCTHKTTGETRRSRRSITLPSHASQKLETRSSTWLESREYSKYLIKVENWILRNPGYALVAAVIG
ZIKV         197  WVVYGTCHHK.KGEARRSRRAVTLPSHSTRKLQTRSQTWLESREYTKHLIKVENWIFRNPGFALVAVAIA
POWV         187  KLEYGRCGR..QAGS.RGKRSVVIPTHAQKDMVGRGHAWLKGDNIRDHVTRVEGWMWKNKLLTAAIVALA
TBEV         189  YLEYGRCGK..QEGS.RTRRSVLIPSHAQGELTGRGHKWLEGDSLRTHLTRVEGWVWKNKLLALAMVTVV
YFV          193  RVAYGKCDS..AGRSRRSRRAIDLPTHENHGLKTRQEKWMTGRMGERQLQKIERWFVRNPFFAVTALTIA
consensus>50      .v.YG.C.........rR..Rsv....H....$..r...W$...........vE.W..rnpgf...a....

DSSP
DENV2        256  YTIGTTHFQRALIFILLTAVTPSMTMRCIGMSNRDFVEGVSGGSWVDIVLEHGSCVTTMAKNKPTLDFEL
DENV1        256  HAIGTSITQKGIIFILLMLVTPSMAMRCVGIGNRDFVEGLSGATWVDVVLEHGSCVTTMAKDKPTLDIEL
DENV3        256  HYIGTSLTQKVVIFILLMLVTPSMTMRCVGVGNRDFVEGLSGATWVDVVLEHGGCVTTMAKNKPTLDIEL
DENV4        255  YMIGQTGIQRTVFFVLMMLVAPSYGMRCVGVGNRDFVEGVSGGAWVDLVLEHGGCVTTMAQGKPTLDFEL
JEV          270  WMLGSNNGQRVVFTILLLLVAPAYSFNCLGMGNRDFIEGASGATWVDLVLEGDSCLTIMANDKPTLDVRM
WNV          266  WMLGSNTMQRVVFVVLLLLVAPAYSFNCLGMSNRDFLEGVSGATWVDLVLEGDSCVTIMSKDKPTIDVKM
SLEV         264  WMLGSNNTQRVVFVIMMLLIAPAYSFNCLGTSNRDFVEGASGATWIDLVLEGGSCVTVMAPEKPTLDFKV
SPOV         265  WTLGSSRSQKIIFVTLLMLVAPAYSIRCIGIGNRDFIEGMSGGTWVDIVLEHGGCVTVMSNDKPTLDFEL
ZIKV         266  WLLGSSTSQKVIYLVMILLIAPAYSIRCIGVSNRDFVEGMSGGTWVDVVLEHGGCVTVMAQDKPTVDIEL
POWV         254  WLMVDSWMARVTVILLALSLGPVYATRCTHLENRDFVTGTQGTTRVSLVLELGGCVTITAEGKPSIDVWL
TBEV         256  WLTLESVVTRVAVLVVLLCLAPVYASRCTHLENRDFVTGTQGTTRVTLVLELGGCVTITAEGKPSMDVWL
YFV          261  YLVGSNMTQRVVIALLVLAVGPAYSAHCIGITDRDFIEGVHGGTWVSATLEQDKCVTVMAPDKPSLDISL
consensus>50      ...g....q.vv..illl.v.P.y...C.g..#RDFveG.sG.tw!d.vLE.g.CvT.ma.dKPtlD..l
```

Data set 1

```
DSSP
DENV2        326  IKTEAKQPATLRKYCIEAKLTNTTTESRCPTQGEPSLNEEQDKRFVCKHSMVDRGWGNGCGLFGKGGIVT
DENV1        326  LKTEVTNPAVLRKLCIEAKISNTTTDSRCPTQGEATLVEEQDTNFVCRRTFVDRGWGNGCGLFGKGSLIT
DENV3        326  QKTEATQLATLRKLCIEGKITNITTDSRCPTQGEAVLPEEQDQNYVCKHTYVDRGWGNGCGLFGKGSLVT
DENV4        325  TKTTAKEVALLRTYCIEASISNITTATRCPTQGEPYLKEEQDQQYICRRDVVDRGWGNGCGLFGKGGVVT
JEV          340  INIEASQLAEVRSYCYHASVTDISTVARCPTTGEAHNEKRADSSYVCKQGFTDRGWGNGCGLFGKGSIDT
WNV          336  MNMEAANLAEVRSYCYLATVSDLSTKAACPTMGEAHNDKRADPAFVCRQGVVDRGWGNGCGLFGKGSIDT
SLEV         334  MKMEATELATVREYCYEATLDTLSTVARCPTTGEAHNTKRSDPTFVCKRDVVDRGWGNGCGLFGKGSIDT
SPOV         335  VTTTASNMAEVRSYCYEANISEMASDSRCPTQGEAYLDKMADSQFVCKRGYVDRGWGNGCGLFGKGSIVT
ZIKV         336  VTTTVSNMAEVRSYCYEASISDMASDSRCPTQGEAYLDKQSDTQYVCKRTLVDRGWGNGCGLFGKGSLVT
POWV         324  EDIFQESPAETREYCLHAKLTNTKVEARCPTTGPATLPEEHQANMVCKRDQSDRGWGNHCGFFGKGSIVA
TBEV         326  DAIYQENPAKTREYCLHAKLSDTKVAARCPTMGPATLAEEHQGGTVCKRDQSDRGWGNHCGLFGKGSIVA
YFV          331  ETVAIDRPAEVRKVCYNAVLTHVKINDKCPSTGEAHLAEENEGDNACKRTYSDRGWGNGCGLFGKGSIVA
consensus>50      ......n.A..R.yC..a...n.....rCPt.Gea.l....#..fvCk....DRGWGNgCGlFGKGsivt
```

```
DSSP
DENV2        396  CAMFRC..KKNMEGKVVQPENLEYTIVITPHSGEE.HAVGNDTG.....KHGKEIKITPQSSITEAELTG
DENV1        396  CAKFKC..VTKLEGKIVQYENLKYSVIVTVHTGDQ.HQVGNETT.....EHGTTATITPQAPTSEIQLTD
DENV3        396  CAKFQC..LEPIEGKVVQYENLKYTVIITVHTGDQ.HQVGNET......QGVTAEITPQASTTEAILPE
DENV4        395  CAKFSC..SGKITGNLVQIENLEYTVVVTVHNGDT.HAVGNDTS.....NHGVTAMITPRSPSVEVKLPD
JEV          410  CAKFSC..TSKAIGRTIQPENIKYEVGIFVHGTTTSENHGNYSA.QVGASQAAKFTITPNAPSITLKLGD
WNV          406  CAKFAC..STKAIGRTILKENIKYEVAIFVHGPTTVESHGNYST.QVGATQAGRLSITPAAPSYTLKLGE
SLEV         404  CAKFTC..KNKATGKTILRENIKYEVAIFVHGSTDSTSHGNYSE.QIGKNQAARFTISPQAPSFTANMGE
SPOV         405  CAKFTC..VKKLTGKSIQPENLEYRVLVSVHASQHGGMINNDTNHQHDKENRARIDITASAPRVEVELGS
ZIKV         406  CAKFTC..SKKMTGKSIQPENLEYRIMLSVHGSQHSGMIGYETD.....EDRAKVEVTPNSPRAEATLGG
POWV         394  CAKFECEEAKKAVGHVVDSTKITYVVKVEPHTGD..YLAANETN.....SNRKSAQFTVASEKVILRLGD
TBEV         396  CVKAACEAKKKATGHVYDANKIVYTVKVEPHTGD..YVAANETH.....SGRKTASFTISSEKTILTMGE
YFV          401  CAKFTC..AKSMSLFEVDQTKIQYVIRAQLHVGAK.QENWN........TDIKTLKFDALSGSQEVEFIG
consensus>50      Cakf.C....k..g..vq.en..Y.!.i..H.........n...............it.........l..
```

```
DSSP
DENV2        458  YGTVTMECSPRTGLDFNEMVLLQMEN......KAWLVHRQWFLDLPLPWLPGADTQGSNWIQKETLVTFK
DENV1        458  YGALTLDCSPRTGLDFNEMVLLTMKK......KSWLVHKQWFLDLPLPWTSGASTSQETWNRQDLLVTFK
DENV3        456  YGTLGLECSPRTGLDFNEMILLTMKN......KAWMVHRQWFFDLPLPWASGATTETPTWNRKELLVTFK
DENV4        457  YGELTLDCEPRSGIDFNEMILMKMKK......KTWLVHKQWFLDLPLPWTAGADTSEVHWNYKERMVTFK
JEV          477  YGEVTLDCEPRSGLNTEAFYVMTVGS......KSFLVHREWFHDLALPWTSPSSTA...WRNRELLMEFE
WNV          473  YGEVTVDCEPRSGIDTNAYYVMTVGT......KTFLVHREWFMDLNLPWSSAGSTV...WRNRETLMEFE
SLEV         471  YGTVTIDCEARSGINTEDYYVFTVKE......KSWLVNRDWFHDLNLPWTSPATTD...WRNRETLVEFE
SPOV         473  FGSFSMECEPRSGLNFGDLYYLTMNN......KHWLVNRDWFHDLSLPWHTGATSNNHHWNNKEALVEFR
ZIKV         469  FGSLGLDCEPRTGLDFSDLYYLTMNN......KHWLVHKEWFHDIPLPWHAGADTGTPHWNNKEALVEFK
POWV         457  YGDVSLTCKVASGIDVAQTVVMSLDSSKDHLPSAWQVHRDWFEDLALPWKHKDNQD...WNSVEKLVEFG
TBEV         459  YGDVSLLCRVASGVDLAQTVILELDKTVEHLPTAWQVHRDWFNDLALPWKHEGAQN...WNNAERLVEFG
YFV          460  YGKATLECQVQTAVDFGNSYIAEMET......ESWIVDRQWAQDLTLPWQSGSGGV...WREMHHLVEFE
consensus>50      %G...ldC..r.g.#..e...l.m........k.wlVhr#Wf.Dl.LPW.........W...e.$v.F.
```

```
DSSP
DENV2        522  NPHAKKQDVVVLGSQEGAMHTALTGATEIQMSSGN....LLFTGHLKCRLRMDKLQLKGMSYSMCTG.KF
DENV1        522  TAHAKKQEVVVLGSQEGAMHTALTGATEIQTSGTT....TIFAGHLKCRLKMDKLILKGMSYVMCTG.SF
DENV3        520  NAHAKKQEVVVLGSQEGAMHTALTGATEIQNSGGT....SIFAGHLKCRLKMDKLELKGMSYAMCTN.TF
DENV4        521  VPHAKRQDVTVLGSQEGAMHSALAGATEVDSGDGN....HMFAGHLKCKVRMEKLRIKGMSYTMCSG.KF
JEV          538  EAHATKQSVVALGSQEGGLHQALAGAIVVEYSSS....VKLTSGHLKCRLKMDKLALKGTTYGMCTE.KF
WNV          534  EPHATKQSVIALGSQEGALHQALAGAIPVEFSSNT...VKLTSGHLKCRVKMEKLQLKGTTYGVCSK.AF
SLEV         532  EPHATKQTVVALGSQEGALHTALAGAIPATVSSST...LTLQSGHLKCRAKLDKVKIKGTTYGMCDS.AF
SPOV         537  EAHAKKQTAVVLGSQEGAVHAALAGALEAESDGHK...ATIYSGHLKCRLKLDKLRLKGMSYALCTG.AF
ZIKV         533  DAHAKRQTVVVLGSQEGAVHTALAGALEAEMDGAK...GRLFSGHLKCRLKMDKLRLKGVSYSLCTA.AF
POWV         524  PPHAVKMDVFNLGDQTAVLLKSLAGVPLASVEGQK...YHLKSGHVTCDVGLEKLKLKGTTYSMCDKAKF
TBEV         526  APHAVKMDVYNLGDQTGVLLKALAGVPVAHIEGTK...YHLKSGHVTCEVGLEKLKMKGLTYTMCDKTKF
YFV          521  PPHATIRVLALGNQEGSLKTALTGAMRVTKDTNDNNLYKLHGGHVSCRVKLSALTLKGTSYKICTD.KM
consensus>50      ..HA.kq.v..LGsQeg.lh.aL.Ga.............l..GHlkCr..$dkl.lKG..Y.mC....f
```

```
DSSP
DENV2        587  KVVKEIAETQHGTIVIRVQYEGDGSPCKIPFEI.MDLEKRHVLGRLITVNPIVTE..KDSPVNIEAEPPF
DENV1        587  KLEKEVAETQHGTVLVQVKYEGTDAPCKIPFSS.QDEKGVTQNGRLITANPIVTD..KEKPVNIEAEPPF
DENV3        585  VLKKEVSETQHGTILIKVEYKGEDAPCKIPFST.EDGQGKAHNGRLITANPVVTK..KEEPVNIEAEPPF
DENV4        586  SIDKEMAETQHGTTVVKVKYEGAGAPCKVPIEI.RDVNKEKVVGRIISSTPLAEN..TNSVTNIELEPPF
JEV          603  SFAKNPADTGHGTVVIELSYSGSDGPCKIPIVSVASLNDMTPVGRLVTVNPFVATSSANSKVLVEMEPPF
WNV          600  KFLGTPADTGHGTVVLELQYTGTDGPCKVPISSVASLNDLTPVGRLVTVNPFVSVATANAKVLIELEPPF
SLEV         598  TFSKNPTDTGHGTVIVELQYTGSNGPCRVPISVTANLMDLTPVGRLVTVNPFISTGGANNKVMIEVEPPF
SPOV         603  TFARTPSETIHGTATVELQYAGEDGPCKVPIVITSDTNSMASTGRLITANPVVTESGANSKMMVEIDPPF
ZIKV         599  TFTKVPAETLHGTVTVEVQYAGTDGPCKIPVQMAVDMQTLTPVGRLITANPVITESTENSKMMLELDPPF
POWV         591  KWKRVPVDSGHDTVVMEVSYTGSDKPCRIPVRAVAHGVPAVNVAMLITPNPTIET...NGGGFIEMQLPP
TBEV         593  TWKRAPTDSGHDTVVMEVTFSGT.KPCRIPVRAVAHGSPDVNVAMLITPNPTIEN...NGGGFIEMQLPP
YFV          590  FFVKNPTDTGHGTVVMQVKVS.KGAPCRIPVIVADDLTAAINKGILVTVNPIAST..NDDEVLIEVNPPF
consensus>50      .......#t.HgTv..e..y.g...PC.!Pi............grl!t.nP.v.....#....iE.#pPf
```

Data set 1

```
DSSP

DENV2_______   654  GDSYIIIGVEPGQLKLNWFKKGSSIGQMFETTMRGAKRMAILGDTAWDFGSLGGVFTSIGKALHQVFGAI
DENV1_______   654  GESYIVVGAGEKALKLSWFKKGSSIGKMFEATARGARRMAILGDTAWDFGSIGGVFTSVGKLIHQIFGTA
DENV3_______   652  GESNIVIGIGDNALKINWYKKGSSIGKMFEATERGARRMAILGDTAWDFGSVGGVLNSLGKMVHQIFGSA
DENV4_______   653  GDSYIVIGVGNSALTLHWFRKGSSIGKMFESTYRGAKRMAILGETAWDFGSVGGLFTSLGKAVHQVFGSV
JEV_________   673  GDSYIVVGRGDKQINHHWHKAGSTLGKAFSTTLKGAQRLAALGDTAWDFGSIGGVFNSIGKAVHQVFGGA
WNV_________   670  GDSYIVVGRGEQQINHHWHKSGSSIGKAFTTTLKGAQRLAALGDTAWDFGSVGGVFTSVGKAVHQVFGGA
SLEV________   668  GDSYIVVGRGTTQINYHWHKEGSSIGKALATTWKGAQRLAVLGDTAWDFGSIGGVFNSIGKAVHQVFGGA
SPOV________   673  GDSYIIVGTGTTKITHHWHRAGSSIGRAFEATMRGAKRMAVLGDTAWDFGSVGGMFNSVGKFVHQVFGSA
ZIKV________   669  GDSYIVIGVGDKKITHHWHRSGSTIGKAFEATVRGAKRMAVLGDTAWDFGSVGGVFNSLGKGIHQIFGAA
POWV________   658  GDNIIYVG....DLSQQWFQKGSTIGRMFEKTRRGLERLSVVGEHAWDFGSVGGVLSSVGKAIHTVLGGA
TBEV________   659  GDNIIYVG....ELSHQWFQKGSSIGRVFQKTKKGIERLTVIGEHAWDFGSAGGFLSSIGKAVHTVLGGA
YFV_________   657  GDSYIIVGRGDSRLTYQWHKEGSSIGKLFTQTMKGVERLAVMGDTAWDFSSAGGFFTSVGKGIHTVFGSA
consensus>50        G#syIv!G.g.......W...GSsiG..f..T..Ga.R$avlG#tAWDFgSvGG.f.SvGK.vHq!fG.a
```

```
DSSP

DENV2_______   724  YGAAFSGVSWTMKILIGVIITWIGMNSRSTSLSVTLVLVGIVTLYLGVMVQADSGCVVSWKNKELKCGSG
DENV1_______   724  YGVLFSGVSWTMKIGIGILLTWLGLNSRSTSLSMTCIAVGMVTLYLGVMVQADSGCVINWKGRELKCGSG
DENV3_______   722  YTALFSGVSWVMKIGIGVLLTWIGLNSKNTSMSFSCIAIGIITLYLGAVVQADMGCVINWKGKELKCGSG
DENV4_______   723  YTTMFGGVSWMIRILIGFLVLWIGTNSRNTSMAMTCIAVGGITLFLGFTVQADMGCVASWSGKELKCGSG
JEV_________   743  FRTLFGGMSWITQGLMGALLLWMGVNARDRSIALAFLATGGVLVFLATNVHADTGCAIDITRKEMRCGSG
WNV_________   740  FRSLFGGMSWITQGLLGALLLWMGINARDRSIALTFLAVGGVLLFLSVNVHADTGCAIDISRQELRCGSG
SLEV________   738  FRTLFGGMSWITQGLLGALLLWMGLQARDRSISLTLLAVGGILIFLATSVQADSGCAIDLQRRELKCGGG
SPOV________   743  FKALFGGMSWFTQLLIGFLLIWMGLNARGGTVAMSFMGIGAMLIFLATSVSGDTGCSVDISRREMRCGSG
ZIKV________   739  FKSLFGGMSWFSQILIGTLLVWLGLNTKNGSISLTCLALGGVMIFLSTAVSADVGCSVDFSKKETRCGTG
POWV________   724  FNTLFGGVGFIPKMLLGVALVWLGLNARNPTMSMTFLAVGALTLMMTMGVGADYGCAIDPERMEIRCGEG
TBEV________   725  FNSIFGGVGFLPKLLLGVALAWLGLNMRNPTMSMSFLLAGGLVLAMTLGVGADVGCAVDTERMELRCGEG
YFV_________   727  FQGLFGGLNWITKVIMGAVLIWVGINTRNMTMSMSMILVGVIMMFLSLGVGADQGCAINFGKRELKCGDG
consensus>50        %..lFgG.sw....l.G..l.W.G.#.rn....m....vG.v..f$...V.aD.GC.id....El.CG.G
```

```
DSSP

DENV2_______   794  IFITDNV
DENV1_______   794  IFVTNEV
DENV3_______   792  IFVTNEV
DENV4_______   793  IFVVDNV
JEV_________   813  IFVHNDV
WNV_________   810  VFIHNDV
SLEV________   808  IFVYNDV
SPOV________   813  IFVYNDV
ZIKV________   809  VFIYNDV
POWV________   794  LVVWKEV
TBEV________   795  LVVWREV
YFV_________   797  IFIFRDS
consensus>50        if!.n#v
```

Data set 2

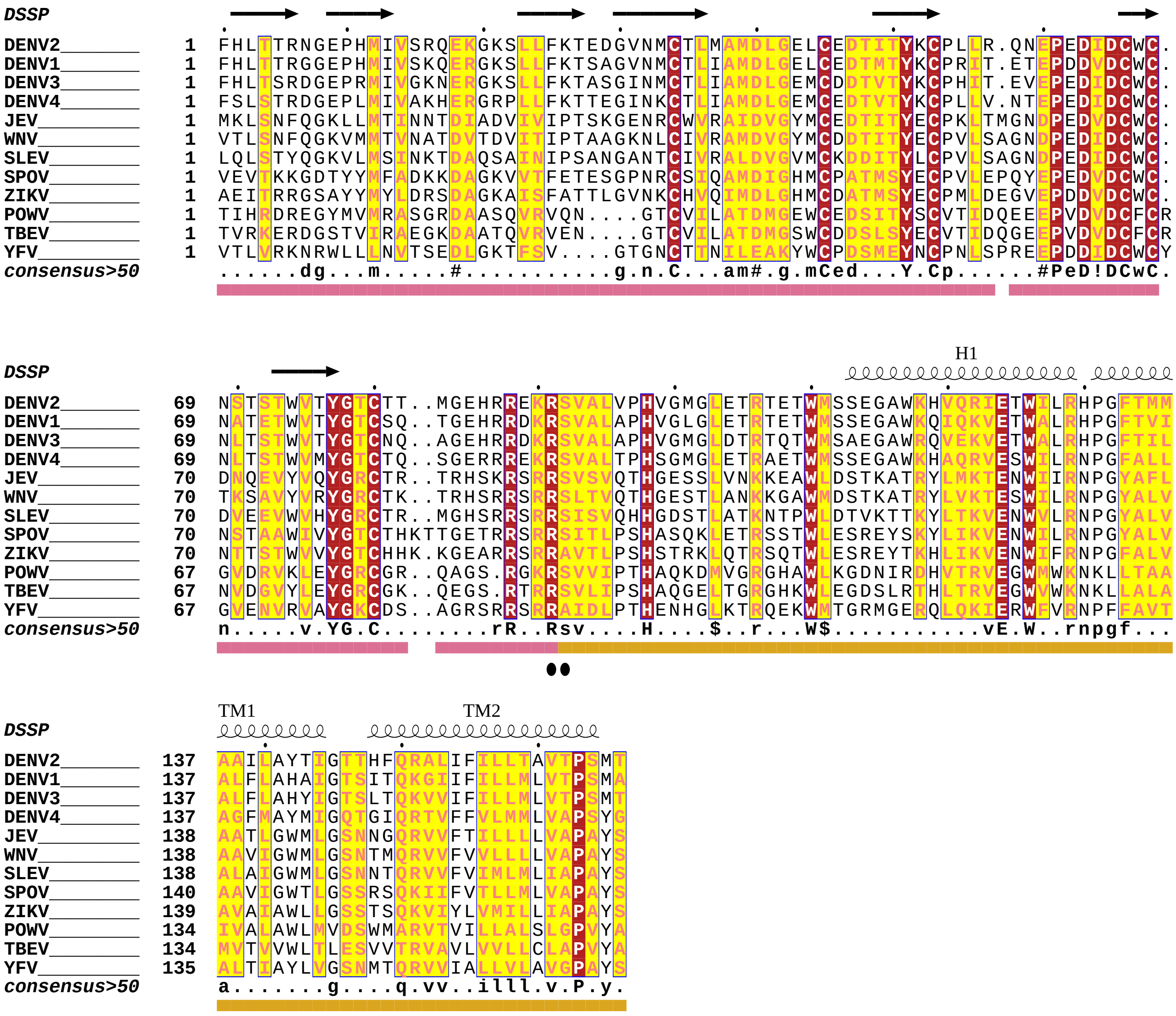
DSSP
DENV2 1 FHLTTRNGEPHMIVSRQEKGKSLLFKTEDGVNMCTLMAMDLGELCEDTITYKCPLLR.QNEPEDIDCWC.
DENV1 1 FHLTTRGGEPHMIVSKQERGKSLLFKTSAGVNMCTLIAMDLGELCEDTMTYKCPRIT.ETEPDDVDCWC.
DENV3 1 FHLTSRDGEPRMIVGKNERGKSLLFKTASGINMCTLIAMDLGEMCDDTVTYKCPHIT.EVEPEDIDCWC.
DENV4 1 FSLSTRDGEPLMIVAKHERGRPLLFKTTEGINKCTLIAMDLGEMCEDTVTYKCPLLV.NTEPEDIDCWC.
JEV 1 MKLSNFQGKLLMTINNTDIADVIVIPTSKGENRCWVRAIDVGYMCEDTITYECPKLTMGNDPEDVDCWC.
WNV 1 VTLSNFQGKVMMTVNATDVTDVITIPTAAGKNLCIVRAMDVGYMCDDTITYECPVLSAGNDPEDIDCWC.
SLEV 1 LQLSTYQGKVLMSINKTDAQSAINIPSANGANTCIVRALDVGVMCKDDITYLCPVLSAGNDPEDIDCWC.
SPOV 1 VEVTKKGDTYYMFADKKDAGKVVTFETESGPNRCSIQAMDIGHMCPATMSYECPVLEPQYEPEDVDCWC.
ZIKV 1 AEITRRGSAYYMYLDRSDAGKAISFATTLGVNKCHVQIMDLGHMCDATMSYECPMLDEGVEPDDVDCWC.
POWV 1 TIHRDREGYMVMRASGRDAASQVRVQN....GTCVILATDMGEWCEDSITYSCVTIDQEEEPVDVDCFCR
TBEV 1 TVRKERDGSTVIRAEGKDAATQVRVEN....GTCVILATDMGSWCDDSLSYECVTIDQGEEPVDVDCFCR
YFV 1 VTLVRKNRWLLLNVTSEDLGKTFSV....GTGNCTTNILEAKYWCPDSMEYNCPNLSPREEPDDIDCWCY
consensus>50 ......dg...m.....#..........g.n.C...am#.g.mCed...Y.Cp......#PeD!DCwC.
H1
DSSP
DENV2 69 NSTSTWVTYGTCTT..MGEHRREKRSVALVPHVGMGLETRTETWMSSEGAWKHVQRIETWILRHPGFTMM
DENV1 69 NATETWVTYGTCSQ..TGEHRRDKRSVALAPHVGLGLETRTETWMSSEGAWKQIQKVETWALRHPGFTVI
DENV3 69 NLTSTWVTYGTCNQ..AGEHRRDKRSVALAPHVGMGLDTRTQTWMSAEGAWRQVEKVETWALRHPGFTIL
DENV4 69 NLTSTWVMYGTCTQ..SGERRREKRSVALTPHSGMGLETRAETWMSSEGAWKHAQRVESWILRNPGFALL
JEV 70 DNQEVYVQYGRCTR..TRHSKRSRRSVSVQTHGESSLVNKKEAWLDSTKATRYLMKTENWIIRNPGYAFL
WNV 70 TKSAVYVRYGRCTK..TRHSRRSRRSLTVQTHGESTLANKKGAWMDSTKATRYLVKTESWILRNPGYALV
SLEV 70 DVEEVWVHYGRCTR..MGHSRRSRRSISVQHHGDSTLATKNTPWLDTVKTTKYLTKVENWVLRNPGYALV
SPOV 70 NSTAAWIVYGTCTHKTTGETRRSRRSITLPSHASQKLETRSSTWLESREYSKYLIKVENWILRNPGYALV
ZIKV 70 NTTSTWVVYGTCHHK.KGEARRSRRAVTLPSHSTRKLQTRSQTWLESREYTKHLIKVENWIFRNPGFALV
POWV 67 GVDRVKLEYGRCGR..QAGS.RGKRSVVIPTHAQKDMVGRGHAWLKGDNIRDHVTRVEGWMWKNKLLTAA
TBEV 67 NVDGVYLEYGRCGK..QEGS.RTRRSVLIPSHAQGELTGRGHKWLEGDSLRTHLTRVEGWVWKNKLLALA
YFV 67 GVENVRVAYGKCDS..AGRSRRSRRAIDLPTHENHGLKTRQEKWMTGRMGERQLQKIERWFVRNPFFAVT
consensus>50 n.....v.YG.C........rR..Rsv....H....$..r...W$..........vE.W..rnpgf...
TM1
TM2
DSSP
DENV2 137 AAILAYTIGTTHFQRALIFILLTAVTPSMT
DENV1 137 ALFLAHAIGTSITQKGIIFILLMLVTPSMA
DENV3 137 ALFLAHYIGTSLTQKVVIFILLMLVTPSMT
DENV4 137 AGFMAYMIGQTGIQRTVFFVLMMLVAPSYG
JEV 138 AATLGWMLGSNNGQRVVFTILLLLVAPAYS
WNV 138 AAVIGWMLGSNTMQRVVFVVLLLLVAPAYS
SLEV 138 ALAIGWMLGSNNTQRVVFVIMLMLIAPAYS
SPOV 140 AAVIGWTLGSSRSQKIIFVTLLMLVAPAYS
ZIKV 139 AVAIAWLLGSSTSQKVIYLVMILLIAPAYS
POWV 134 IVALAWLMVDSWMARVTVILLALSLGPVYA
TBEV 134 MVTVVWLTLESVVTRVAVLVVLLCLAPVYA
YFV 135 ALTIAYLVGSNMTQRVVIALLVLAVGPAYS
consensus>50 a.......g....q.vv..illl.v.P.y.

Data set 3

```
DSSP

DENV2________    1  MRCIGMSNRDFVEGVSGGSWVDIVLEHGSCVTTMAKNKPTLDFELIKTEAKQPATLRKYCIEAKLTNTTT
DENV1________    1  MRCVGIGNRDFVEGLSGATWVDVVLEHGSCVTTMAKDKPTLDIELLKTEVTNPAVLRKLCIEAKISNTTT
DENV3________    1  MRCVGVGNRDFVEGLSGATWVDVVLEHGGCVTTMAKNKPTLDIELQKTEATQLATLRKLCIEGKITNITT
DENV4________    1  MRCVGVGNRDFVEGVSGGAWVDLVLEHGGCVTTMAQGKPTLDFELTKTTAKEVALLRTYCIEASISNITT
JEV__________    1  FNCLGMGNRDFIEGASGATWVDLVLEGDSCLTIMANDKPTLDVRMINIEASQLAEVRSYCYHASVTDIST
WNV__________    1  FNCLGMSNRDFLEGVSGATWVDLVLEGDSCVTIMSKDKPTIDVKMMNMEAANLAEVRSYCYLATVSDLST
SLEV_________    1  FNCLGTSNRDFVEGASGATWIDLVLEGGSCVTVMAPEKPTLDFKVMKMEATELATVREYCYEATLDTLST
SPOV_________    1  IRCIGIGNRDFIEGMSGGTWVDIVLEHGGCVTVMSNDKPTLDFELVTTTASNMAEVRSYCYEANISEMAS
ZIKV_________    1  IRCIGVSNRDFVEGMSGGTWVDVVLEHGGCVTVMAQDKPTVDIELVTTTVSNMAEVRSYCYEASISDMAS
POWV_________    1  TRCTHLENRDFVTGTQGTTRVSLVLELGGCVTITAEGKPSIDVWLEDIFQESPAETREYCLHAKLTNTKV
TBEV_________    1  SRCTHLENRDFVTGTQGTTRVTLVLELGGCVTITAEGKPSMDVWLDAIYQENPAKTREYCLHAKLSDTKV
YFV__________    1  AHCIGITDRDFIEGVHGGTWVSATLEQDKCVTVMAPDKPSLDISLETVAIDRPAEVRKVCYNAVLTHVKI
consensus>50        ..C.g..#RDFveG.sG.tw!d.vLE.g.CvT.ma.dKPtlD..l......n.A..R.yC..a...n...

DSSP

DENV2________   71  ESRCPTQGEPSLNEEQDKRFVCKHSMVDRGWGNGCGLFGKGGIVTCAMFRC..KKNMEGKVVQPENLEYT
DENV1________   71  DSRCPTQGEATLVEEQDTNFVCRRTFVDRGWGNGCGLFGKGSLITCAKFKC..VTKLEGKIVQYENLKYS
DENV3________   71  DSRCPTQGEAVLPEEQDQNYVCKHTYVDRGWGNGCGLFGKGSLVTCAKFQC..LEPIEGKVVQYENLKYT
DENV4________   71  ATRCPTQGEPYLKEEQDQQYICRRDVVDRGWGNGCGLFGKGGVVTCAKFSC..SGKITGNLVQIENLEYT
JEV__________   71  VARCPTTGEAHNEKRADSSYVCKQGFTDRGWGNGCGLFGKGSIDTCAKFSC..TSKAIGRTIQPENIKYE
WNV__________   71  KAACPTMGEAHNEKRADPAFVCRQGVVDRGWGNGCGLFGKGSIDTCAKFAC..STKAIGRTILKENIKYE
SLEV_________   71  VARCPTTGEAHNTKRSDPTFVCKRDVVDRGWGNGCGLFGKGSIDTCAKFTC..KNKATGKTILRENIKYE
SPOV_________   71  DSRCPTQGEAYLDKMADSQFVCKRGYVDRGWGNGCGLFGKGSIVTCAKFTC..VKKLTGKSIQPENLEYR
ZIKV_________   71  DSRCPTQGEAYLDKQSDTQYVCKRTLVDRGWGNGCGLFGKGSLVTCAKFTC..SKKMTGKSIQPENLEYR
POWV_________   71  EARCPTTGPATLPEEHQANMVCKRDQSDRGWGNHCGFFGKGSIVACVKAACEAKKKAVGHVYDSTKITYV
TBEV_________   71  AARCPTMGPATLAEEHQGGTVCKRDQSDRGWGNHCGLFGKGSIVACVKAACEAKKKATGHVYDANKIVYT
YFV__________   71  NDKCPSTGEAHLAEENEGDNACKRTYSDRGWGNGCGLFGKGSIVACAKFTC..AKSMSLFEVDQTKIQYV
consensus>50        ..rCPt.Gea.l....#..fvCk....DRGWGNgCGlFGKGsivtCakf.C....k..g..vq.en..Y.

DSSP

DENV2________  139  IVITPHSGEE.HAVGNDTG.....KHGKEIKITPQSSITEAELTGYGTVTMECSPRTGLDFNEMVLLQME
DENV1________  139  VIVTVHTGDQ.HQVGNETT.....EHGTTATITPQAPTSEIQLTDYGALTLDCSPRTGLDFNEMVLLTMK
DENV3________  139  VIITVHTGDQ.HQVGNET.......QGVTAEITPQASTTEAILPEYGTLGLECSPRTGLDFNEMILLTMK
DENV4________  139  VVVTVHNGDT.HAVGNDTS.....NHGVTAMITPRSPSVEVKLPDYGELTLDCEPRSGIDFNEMILMKMK
JEV__________  139  VGIFVHGTTTSENHGNYSA.QVGASQAAKFTITPNAPSITLKLGDYGEVTLDCEPRSGLNTEAFYVMTVG
WNV__________  139  VAIFVHGPTTVESHGNYST.QVGATQAGRLSITPAAPSYTLKLGEYGEVTVDCEPRSGIDTNAYYVMTVG
SLEV_________  139  VAIFVHGSTDSTSHGNYSE.QIGKNQAARFTISPQAPSFTANMGEYGTVTIDCEARSGINTEDYYVFTVK
SPOV_________  139  VLVSVHASQHGGMINNDTNHQHDKENRARIDITASAPRVEVELGSFGSFSMECEPRSGLNFGDLYYLTMN
ZIKV_________  139  IMLSVHGSQHSGMIGYETD.....EDRAKVEVTPNSPRAEATLGGFGSLGLDCEPRTGLDFSDLYYLTMN
POWV_________  141  VKVEPHTGD..YLAANETN.....SNRKSAQFTVASEKVILRLGDYGDVSLTCKVASGIDVAQTVVMSLD
TBEV_________  141  VKVEPHTGD..YVAANETH.....SGRKTASFTISSEKTILTMGEYGDVSLLCRVASGVDLAQTVILELD
YFV__________  139  IRAQLHVGAK.QENWN........TDIKTLKFDALSGSQEVEFIGYGKATLECQVQTAVDFGNSYIAEME
consensus>50        !.i..H.........n..............it.........l..%G...ldC..r.g.#..e...l.m.

DSSP

DENV2________  203  N......KAWLVHRQWFLDLPLPWLPGADTQGSNWIQKETLVTFKNPHAKKQDVVVLGSQEGAMHTALTG
DENV1________  203  K......KSWLVHKQWFLDLPLPWTSGASTSQETWNRQDLLVTFKTAHAKKQEVVVLGSQEGAMHTALTG
DENV3________  201  N......KAWMVHRQWFFDLPLPWASGATTETPTWNRKELLVTFKNAHAKKQEVVVLGSQEGAMHTALTG
DENV4________  203  K......KTWLVHKQWFLDLPLPWTAGADTSEVHWNYKERMVTFKVPHAKRQDVTVLGSQEGAMHSALAG
JEV__________  208  S......KSFLVHREWFHDLALPWTSPSSTA...WRNRELLMEFEEAHATKQSVVALGSQEGGLHQALAG
WNV__________  208  T......KTFLVHREWFMDLNLPWSSAGSTV...WRNRETLMEFEEPHATKQSVIALGSQEGALHQALAG
SLEV_________  208  E......KSWLVNRDWFHDLNLPWTSPATTD...WRNRETLVEFEEPHATKQTVVALGSQEGALHTALAG
SPOV_________  209  N......KHWLVNRDWFHDLSLPWHTGATSNNHHWNNKEALVEFREAHAKKQTAVVLGSQEGAVHAALAG
ZIKV_________  204  N......KHWLVHKEWFHDIPLPWHAGADTGTPHWNNKEALVEFKDAHAKRQTVVVLGSQEGAVHTALAG
POWV_________  204  SSKDHLPSAWQVHRDWFEDLALPWKHKDNQD...WNSVEKLVEFGPPHAVKMDVFNLGDQTAVLLKSLAG
TBEV_________  204  KTVEHLPTAWQVHRDWFNDLALPWKHEGAQN...WNNAERLVEFGAPHAVKMDVYNLGDQTGVLLKALAG
YFV__________  200  T......ESWIVDRQWAQDLTLPWQSGSGGV...WREMHHLVEFEPPHAATIRVLALGNQEGSLKTALTG
consensus>50        .......k.wlVhr#Wf.Dl.LPW.........W...e.$v.F...HA.kq.v..LGsQeg.lh.aL.G

DSSP

DENV2________  267  ATEIQMSSGN....LLFTGHLKCRLRMDKLQLKGMSYSMCTG.KFKVVKEIAETQHGTIVIRVQYEGDGS
DENV1________  267  ATEIQTSGTT....TIFAGHLKCRLKMDKLILKGMSYVMCTG.SFKLEKEVAETQHGTVLVQVKYEGTDA
DENV3________  265  ATEIQNSGGT....SIFAGHLKCRLKMDKLELKGMSYAMCTN.TFVLKKEVSETQHGTILIKVEYKGEDA
DENV4________  267  ATEVDSGDGN....HMFAGHLKCKVRMEKLRIKGMSYTMCSG.KFSIDKEMAETQHGTVVVKVKYEGAGA
JEV__________  269  AIVVEYSSS....VKLTSGHLKCRLKMDKLALKGTTYGMCTE.KFSFAKNPADTGHGTVVIELSYSGSDG
WNV__________  269  AIPVEFSSNT...VKLTSGHLKCRVKMEKLQLKGTTYGVCSK.AFKFLGTPADTGHGTVVLELQYTGTDG
SLEV_________  269  AIPATVSSST...LTLQSGHLKCRAKLDKVKIKGTTYGMCDS.AFTFSKNPTDTGHGTVIVELQYTGSNG
SPOV_________  273  ALEAESDGHK...ATIYSGHLKCRLKLDKLRLKGMSYALCTG.AFTFARTPSETIHGTATVELQYAGEDG
ZIKV_________  268  ALEAEMDGAK...GRLFSGHLKCRLKMDKLRLKGVSYSLCTA.AFTFTKVPAETLHGTVTVEVQYAGTDG
POWV_________  271  VPLASVEGQK...YHLKSGHVTCDVGLEKLKLKGTTYSMCDKAKFKWKRVPVDSGHDTVVMEVSYTGSDK
TBEV_________  271  VPVAHIEGTK...YHLKSGHVTCEVGLEKLKMKGLTYTMCDKTKFTWKRAPTDSGHDTVVMEVTFSGT.K
YFV__________  261  AMRVTKDTNDNNLYKLHGGHVSCRVKLSALTLKGTSYKICTD.KMFFVKNPTDTGHGTVVMQVKVS.KGA
consensus>50        a.............l..GHlkCr..$dkl.lKG..Y.mC....f.......#t.HgTv..e..y.g...
```

Data set 3

```
DSSP
DENV2_______   332  PCKIPFEI.MDLEKRHVLGRLITVNPIVTE..KDSPVNIEAEPPFGDSYIIIGVEPGQLKLNWFKKGSSI
DENV1_______   332  PCKIPFSS.QDEKGVTQNGRLITANPIVTD..KEKPVNIEAEPPFGESYIVVGAGEKALKLSWFKKGSSI
DENV3_______   330  PCKIPFST.EDGQGKAHNGRLITANPVVTK..KEEPVNIEAEPPFGESNIVIGIGDNALKINWYKKGSSI
DENV4_______   332  PCKVPIEI.RDVNKEKVVGRIISSTPLAEN..TNSVTNIELEPPFGDSYIVIGVGNSALTLHWFRKGSSI
JEV_________   334  PCKIPIVSVASLNDMTPVGRLVTVNPFVATSSANSKVLVEMEPPFGDSYIVVGRGDKQINHHWHKAGSTL
WNV_________   335  PCKVPISSVASLNDLTPVGRLVTVNPFVSVATANAKVLIELEPPFGDSYIVVGRGEQQINHHWHKSGSSI
SLEV________   335  PCRVPISVTANLMDLTPVGRLVTVNPFISTGGANNKVMIEVEPPFGDSYIVVGRGTTQINYHWHKEGSSI
SPOV________   339  PCKVPIVITSDTNSMASTGRLITANPVVTESGANSKMMVEIDPPFGDSYIVIGTGTTKITHHWHRAGSSI
ZIKV________   334  PCKIPVQMAVDMQTLTPVGRLITANPVITESTENSKMMLELDPPFGDSYIVIGVGDKKITHHWHRSGSTI
POWV________   338  PCRIPVRAVAHGVPAVNVAMLITPNPTIET...NGGGFIEMQLPPGDNIIYVG....DLSQQWFQKGSTI
TBEV________   337  PCRIPVRAVAHGSPDVNVAMLITPNPTIEN...NGGGFIEMQLPPGDNIIYVG....ELSHQWFQKGSSI
YFV_________   329  PCRIPVIVADDLTAAINKGILVTVNPIAST..NDDEVLIEVNPPFGDSYIIVGRGDSRLTYQWHKEGSSI
consensus>50        PC.!Pi............grl!t.nP.v.....#....iE.#pPfG#syIv!G.g.......W...GSsi

                    H1                       H2                    TM1
DSSP
DENV2_______   399  GQMFETTMRGAKRMAILGDTAWDFGSLGGVFTSIGKALHQVFGAIYGAAFSGVSWTMKILIGVIITWIGM
DENV1_______   399  GKMFEATARGARRMAILGDTAWDFGSIGGVFTSVGKLIHQIFGTAYGVLFSGVSWTMKIGIGILLTWLGL
DENV3_______   397  GKMFEATERGARRMAILGDTAWDFGSVGGVLNSLGKMVHQIFGSAYTALFSGVSWVMKIGIGVLLTWIGL
DENV4_______   399  GKMFESTYRGAKRMAILGETAWDFGSVGGLFTSLGKAVHQVFGSVYTTMFGGVSWMIRILIGFLVLWIGT
JEV_________   404  GKAFSTTLKGAQRLAALGDTAWDFGSIGGVFNSIGKAVHQVFGGAFRTLFGGMSWITQGLMGALLLWMGV
WNV_________   405  GKAFTTTLKGAQRLAALGDTAWDFGSVGGVFTSVGKAVHQVFGGAFRSLFGGMSWITQGLLGALLLWMGI
SLEV________   405  GKALATTWKGAQRLAVLGDTAWDFGSIGGVFNSIGKAVHQVFGGAFRTLFGGMSWITQGLLGALLLWMGL
SPOV________   409  GRAFEATMRGAKRMAVLGDTAWDFGSVGGMFNSVGKFVHQVFGSAFKALFGGMSWFTQLLIGFLLIWMGL
ZIKV________   404  GKAFEATVRGAKRMAVLGDTAWDFGSVGGVFNSLGKGIHQIFGAAFKSLFGGMSWFSQILIGTLLVWLGL
POWV________   401  GRMFEKTRRGLERLSVVGEHAWDFGSVGGVLSSVGKAIHTVLGGAFNTLFGGVGFIPKMLLGVALVWLGL
TBEV________   400  GRVFQKTKKGIERLTVIGEHAWDFGSAGGFLSSIGKAVHTVLGGAFNSIFGGVGFLPKLLLGVALAWLGL
YFV_________   397  GKLFTQTMKGVERLAVMGDTAWDFSSAGGFFTSVGKGIHTVFGSAFQGLFGGLNWITKVIMGAVLIWVGI
consensus>50        G..f..T..Ga.R$avlG#tAWDFgSvGG.f.SvGK.vHq!fG.a%..lFgG.sw....l.G..l.W.G.

                          TM2
DSSP
DENV2_______   469  NSRSTSLSVTLVLVGIVTLYLGVMVQA
DENV1_______   469  NSRSTSLSMTCIAVGMVTLYLGVMVQA
DENV3_______   467  NSKNTSMSFSCIAIGIITLYLGAVVQA
DENV4_______   469  NSRNTSMAMTCIAVGGITLFLGFTVQA
JEV_________   474  NARDRSIALAFLATGGVLVFLATNVHA
WNV_________   475  NARDRSIALTFLAVGGVLLFLSVNVHA
SLEV________   475  QARDRSISLTLLAVGGILIFLATSVQA
SPOV________   479  NARGGTVAMSFMGIGAMLIFLATSVSG
ZIKV________   474  NTKNGSISLTCLALGGVMIFLSTAVSA
POWV________   471  NARNPTMSMTFLAVGALTLMMTMGVGA
TBEV________   470  NMRNPTMSMSFLLAGGLVLAMTLGVGA
YFV_________   467  NTRNMTMSMSMILVGVIMMFLSLGVGA
consensus>50        #.rn....m....vG.v..f$...V.a
```

Data set 4

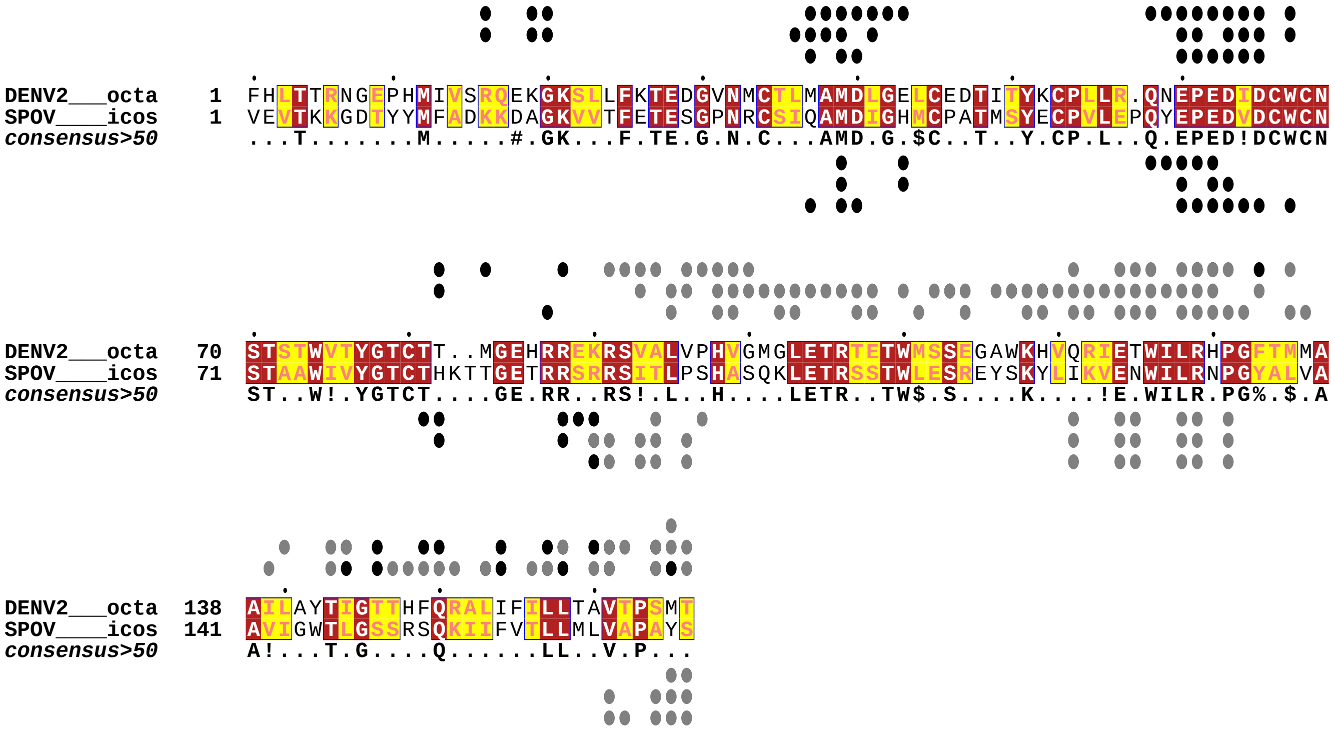
DENV2___octa 1 FHLTTRNGEPHMIVSRQEKGKSLLFKTEDGVNMCTLMAMDLGELCEDTITYKCPLLR.QNEPEDIDCWCN
SPOV____icos 1 VEVTKKGDTYYMFADKKDAGKVVTFETESGPNRCSIQAMDIGHMCPATMSYECPVLEPQYEPEDVDCWCN
consensus>50 ...T.......M.....#.GK...F.TE.G.N.C...AMD.G.$C..T..Y.CP.L..Q.EPED!DCWCN
DENV2___octa 70 STSTWVTYGTCTT..MGEHRREKRSVALVPHVGMGLETRTETWMSSEGAWKHVQRIETWILRHPGFTMMA
SPOV____icos 71 STAAWIVYGTCTHKTTGETRRSRRSITLPSHASQKLETRSSTWLESREYSKYLIKVENWILRNPGYALVA
consensus>50 ST..W!.YGTCT....GE.RR..RS!.L..H....LETR..TW$.S....K....!E.WILR.PG%.$.A
DENV2___octa 138 AILAYTIGTTHFQRALIFILLTAVTPSMT
SPOV____icos 141 AVIGWTLGSSRSQKIIFVTLLMLVAPAYS
consensus>50 A!...T.G....Q......LL..V.P...

Data set 5

```
DENV2___octa     1  MRCIGMSNRDFVEGVSGGSWVDIVLEHGSCVTTMAKNKPTLDFELIKTEAKQPATLRKYCIEAKLTNTTT
SPOV____icos     1  IRCIGIGNRDFIEGMSGGTWVDIVLEHGGCVTVMSNDKPTLDFELVTTTASNMAEVRSYCYEANISEMAS
consensus>50        .RCIG..NRDF!EG.SGG.WVDIVLEHG.CVT.M..#KPTLDFEL!.T.A.#.A..R.YC.EA...#...

DENV2___octa    71  ESRCPTQGEPSLNEEQDKRFVCKHSMVDRGWGNGCGLFGKGGIVTCAMFRCKKNMEGKVVQPENLEYTIV
SPOV____icos    71  DSRCPTQGEAYLDKMADSQFVCKRGYVDRGWGNGCGLFGKGSIVTCAKFTCVKKLTGKSIQPENLEYRVL
consensus>50        #SRCPTQGE..L#...D..FVCK...VDRGWGNGCGLFGKG.IVTCA.F.C.K.$.GK.!QPENLEY.!.

DENV2___octa   141  ITPHSGEE.HAVGNDTG.....KHGKEIKITPQSSITEAELTGYGTVTMECSPRTGLDFNEMVLLQMENK
SPOV____icos   141  VSVHASQHGGMINNDTNHQHDKENRARIDITASAPRVEVELGSFGSFSMECEPRSGLNFGDLYYLTMNNK
consensus>50        !..H..#....!.NDT...........I.IT......E.EL..%G...MEC.PR.GL#F.#$..L.M#NK

DENV2___octa   205  AWLVHRQWFLDLPLPWLPGADTQGSNWIQKETLVTFKNPHAKKQDVVVLGSQEGAMHTALTGATEIQMSS
SPOV____icos   211  HWLVNRDWFHDLSLPWHTGATSNNHHWNNKEALVEFREAHAKKQTAVVLGSQEGAVHAALAGALEAESDG
consensus>50        .WLV.R#WF.DL.LPW..GA..#...W.#KE.LV.F.#.HAKKQ..VVLGSQEGA.H.AL.GA.E.#...

DENV2___octa   275  GN.LLFTGHLKCRLRMDKLQLKGMSYSMCTGKFKVVKEIAETQHGTIVIRVQYEGDGSPCKIPFEI.MDL
SPOV____icos   281  HKATIYSGHLKCRLKLDKLRLKGMSYALCTGAFTFARTPSETIHGTATVELQYAGEDGPCKVPIVITSDT
consensus>50        .....%.GHLKCRL.$DKL.LKGMSY.$CTG.F.......ET.HGT..!..QY.G#..PCK!P..I..D.

DENV2___octa   343  EKRHVLGRLITVNPIVTE..KDSPVNIEAEPPFGDSYIIIGVEPGQLKLNWFKKGSSIGQMFETTMRGAK
SPOV____icos   351  NSMASTGRLITANPVVTESGANSKMMVEIDPPFGDSYIIVGTGTTKITHHWHRAGSSIGRAFEATMRGAK
consensus>50        #.....GRLIT.NP!VTE...#S...!E.#PPFGDSYII!G.........W...GSSIG..FE.TMRGAK

DENV2___octa   411  RMAILGDTAWDFGSLGGVFTSIGKALHQVFGAIYGAAFSGVSWTMKILIGVIITWIGMNSRSTSLSVTLV
SPOV____icos   421  RMAVLGDTAWDFGSVGGMFNSVGKFVHQVFGSAFKALFGGMSWFTQLLIGFLLIWMGLNARGGTVAMSFM
consensus>50        RMA!LGDTAWDFGS.GG.F.S!GK..HQVFG..%.A.F.G.SW....LIG....W.G$N.R.........

DENV2___octa   481  LVGIVTLYLGVMVQA
SPOV____icos   491  GIGAMLIFLATSVSG
consensus>50        .!G....%L...V..
```
